# Surface Engineering Strategy Using Urea To Improve the Rate Performance of Na_2_Ti_3_O_7_ in Na‐Ion Batteries

**DOI:** 10.1002/chem.202003129

**Published:** 2021-01-14

**Authors:** Sara I. R. Costa, Yong‐Seok Choi, Alistair J. Fielding, Andrew J. Naylor, John M. Griffin, Zdeněk Sofer, David O. Scanlon, Nuria Tapia‐Ruiz

**Affiliations:** ^1^ Department of Chemistry Lancaster University Lancaster LA1 4YB UK; ^2^ The Faraday Institution Harwell Campus Didcot OX11 0RA UK; ^3^ Department of Chemistry University College London 20 Gordon Street London WC1H 0AJ UK; ^4^ Thomas Young Centre University College London Gower Street London WC1E 6BT UK; ^5^ School of Pharmacy and Biomolecular Sciences Liverpool John Moores University Liverpool L3 3AF UK; ^6^ Department of Chemistry—Ångström Laboratory Uppsala University Box 538 75121 Uppsala Sweden; ^7^ Department of Inorganic Chemistry University of Chemistry and Technology Prague Technická 5 16628 Prague 6 Czech Republic; ^8^ Diamond Light Source Ltd. Diamond House Harwell Science and Innovation Campus Didcot Oxfordshire OX11 0DE UK

**Keywords:** anode, Na_2_Ti_3_O_7_ and Na_2_Ti_6_O_13_, oxygen vacancies, sodium titanate, sodium-ion batteries, urea

## Abstract

Na_2_Ti_3_O_7_ (NTO) is considered a promising anode material for Na‐ion batteries due to its layered structure with an open framework and low and safe average operating voltage of 0.3 V vs. Na^+^/Na. However, its poor electronic conductivity needs to be addressed to make this material attractive for practical applications among other anode choices. Here, we report a safe, controllable and affordable method using urea that significantly improves the rate performance of NTO by producing surface defects such as oxygen vacancies and hydroxyl groups, and the secondary phase Na_2_Ti_6_O_13_. The enhanced electrochemical performance agrees with the higher Na^+^ ion diffusion coefficient, higher charge carrier density and reduced bandgap observed in these samples, without the need of nanosizing and/or complex synthetic strategies. A comprehensive study using a combination of diffraction, microscopic, spectroscopic and electrochemical techniques supported by computational studies based on DFT calculations, was carried out to understand the effects of this treatment on the surface, chemistry and electronic and charge storage properties of NTO. This study underscores the benefits of using urea as a strategy for enhancing the charge storage properties of NTO and thus, unfolding the potential of this material in practical energy storage applications.

## Graphical abstract

**A safe, controllable and affordable** method using urea at mild temperatures to synthesise a series of sodium titanate samples with different levels of oxygen vacancies is reported. The formation of oxygen vacancies leads to the reduction of Ti^4+^ to Ti^3+^ ions in NTO, together with the formation of hydroxyl groups and a secondary phase, Na_2_Ti_6_O_13_.The urea‐treated samples showed superior electrochemical performance at high rates with respect to pristine NTO.

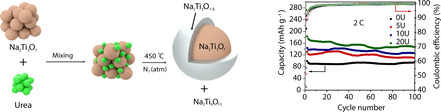

## Introduction

The increasing demand for electrochemical energy storage devices has resulted in rapid development and utilisation of Li‐ion batteries (LIBs) in recent years.^[1]^ LIBs are widely used in portable electronic devices, electric/hybrid vehicles and smart grid systems.^[1]^ However, the scarcity of lithium sources combined with their growing demand have motivated the development of alternative storage technologies.^[2]^ Sodium‐ion batteries (SIBs) offer a promising low‐cost energy storage alternative to LIBs owing to the abundance of sodium sources on Earth.^[3]^ Furthermore, inexpensive aluminium current collectors can be used on the anode side, instead of the more expensive copper in LIBs.^[4]^ Nevertheless, one of the greatest challenges related to the full incorporation of SIBs into the market is finding suitable and safe anode materials that deliver high and stable capacities at low voltages in classic organic electrolytes.^[4]^

Titanium‐based materials arise as one of the most promising candidates due to their non‐toxicity, large abundance and low manufacturing cost.^[5]^ Among this family of compounds, there has been a growing interest in exploring the zig‐zag layered Na_2_Ti_3_O_7_ (NTO) phase as a SIB anode material in the last decade.^[6]^ NTO was first tested as an anode in SIBs about a decade ago, where it was shown to reversibly intercalate up to 2 Na ions per formula, resulting in a high theoretical capacity (177 mAh g^−1^) at a low and safe average potential of 0.3 V vs. Na^+^/Na.^[6]^ The insertion of Na^+^ ions in NTO is commonly accepted to proceed through a two‐phase reaction mechanism that leads to the formation of the end‐discharge product Na_4_Ti_3_O_7_ (with the corresponding reduction of 2/3 Ti^4+^ ions to Ti^3+^).[^6^, ^7^, ^8^] More recent studies have shown a stable and partially sodiated intermediate phase with composition Na_3−*x*_Ti_3_O_7_ which forms upon the first discharge process.^[7]^ Unfortunately, the prospects for practical application of NTO in SIBs are compromised by sluggish Na^+^ ion diffusion kinetics due to its structural distortion upon uptake of Na^+^ ions and the intrinsically electronically insulating nature of NTO, associated with a large bandgap of 3.7–3.9 eV.[^3^, ^9^] These result in poor electrochemical performance at high charge/discharge rates, limiting the use of NTO in high power applications.[^3^, ^10^] Several research strategies including carbon‐composite fabrication,[^7^, ^8^, ^11^] nanostructuring,[^12^, ^13^, ^14^, ^15^, ^16^] doping[^17^, ^18^] and surface defect engineering[^9^, ^10^, ^19^, ^20^] have shown to improve the electrical/ionic conductivity and electrochemical performance of NTO. Surface defect engineering typically involves the introduction of oxygen vacancies in the NTO structure, which act as n‐type defects, improving charge transfer processes by narrowing the bandgap. Typically, synthetic methods that introduce oxygen vacancies in NTO involve the direct use of H_2_ (which may raise safety concerns), limiting their practical applications.[^9^, ^10^] Therefore, considering the potential benefits associated to the creation of oxygen‐deficient materials, it is desirable to develop a safe, controllable and affordable synthesis method to obtain NTO with improved electrochemical performance through an effective surface engineering method. In this context, urea (CH_4_N_2_O) has been successfully used as a reducing agent to create oxygen vacancies in metal oxide photocatalysts such as WO_3_ and BiOBr.[^21^, ^22^] At relatively mild temperatures, urea decomposes into ammonia which further decomposes to produce reactive H_2_ that removes oxygen from the structure.^[21]^ Herein, we have prepared NTO with different levels of oxygen vacancies by mixing as‐synthesised NTO with urea at different concentrations (5, 10 and 20 wt.%) before annealing in a N_2_ atmosphere at 450 °C for 2 h. The 20 wt. % urea sample was found to be the optimal concentration, showing initial discharge capacities of 316 mAh g^−1^ (1 C) and 272 mAh g^−1^ (2 C) when tested as an anode in Na‐ion half‐cells. After 100 cycles, discharge capacities of 154 mAh g^−1^ (1 C) and 145 mAh g^−1^ (2 C) were obtained, which are significantly higher than those observed in the pristine material 106 mAh g^−1^ (1 C) and 90 mAh g^−1^ (2 C)). The improved electrochemical performance is attributed to the higher Na^+^ ion diffusion coefficient, higher charge carrier density and reduced bandgap observed in the urea‐treated sample. As will be described later, the urea treatment not only produces oxygen vacancies but also leads to the formation of Ti^3+^ and Ti−OH species and Na_2_Ti_6_O_13_, which together are responsible for the improved rate performance. Our work will examine the effects of the urea treatment on the surface, chemistry and electronic and charge storage properties of NTO, with a combination of experimental characterisation techniques and computational studies based on density functional theory (DFT).

## Experimental Section

**Synthesis of Na_2_Ti_3_O_7_**: NTO was synthesised by a solid‐state reaction method using a stoichiometric mixture of TiO_2_ (Fisher Scientific, 98 %) and anhydrous Na_2_CO_3_ (Sigma–Aldrich, 99.9 %). These were mixed in a planetary ball mill at 400 rpm, followed by a heat treatment at 800 °C for 20 h. The synthesised NTO powders were mixed with different concentrations of urea, CH_4_N_2_O, (Thermoscientific, 99.5 %) (5, 10 and 20 wt %), followed by an annealing treatment at 450 °C for 2 h under an N_2_ atmosphere. The obtained powders, herein described as 0U, 5U, 10U and 20U, correspond to NTO mixed with 0, 5, 10 and 20 wt. % urea, respectively. The as‐prepared powders were mixed with 50 wt % sucrose, C_12_H_22_O_11_, (Sigma–Aldrich, 99.5 %) and then subjected to a pyrolysis treatment at 700 °C for 5 h under flowing argon to carbon‐coat the powders to allow for a good comparison between the pristine material and the urea‐treated samples while improving the overall electronic conductivity of the samples.^[8]^

**Physicochemical characterisation**: Powder X‐ray diffraction (PXRD) data were recorded at room temperature using a Smartlab diffractometer (Rigaku Corporation) equipped with a 9 kW Cu rotating anode (*λ*=1.54056 Å) operating in reflection mode with Bragg–Brentano geometry. Data were collected in the 5–70 ° 2*θ* range at a scan speed of 0.02 ° s^−1^. The NTO structure was refined against powder X‐ray diffraction data using the Rietveld method, with the GSAS‐EXPGUI software interface.[^23^, ^24^] The peak shapes were modelled with a Gaussian–Lorentzian function and the background, lattice parameters, atomic positions and thermal parameters were refined. The thermal parameters for individual Na, Ti and O atoms were refined isotropically and constrained to be identical. The occupancy for all atoms was fixed to *n*=1.

The microstructure of the samples was examined using a field emission scanning electron microscope (FESEM; JEOL JSM‐7800F) operated at 5 kV and 5 mA. Energy‐dispersive X‐ray analysis (EDX) was carried out at 20 kV to assess the elemental composition, using the AZtecEnergy software. Before the analysis, powders were coated with a uniform layer of Au/Pd by sputtering deposition to provide surface conductivity and prevent surface charging.

Transmission electron microscopy (TEM, JEOL 2200FE) was carried out at 100 keV to assess the morphology and thickness of the carbon coating layer after the pyrolysis treatment. Initially, samples were prepared by mixing the as‐prepared powders with dried acetonitrile using an ultrasonication method. The suspension was then drop cast onto a TEM grid inside the glovebox, dried under vacuum and transferred to the microscope under an Ar atmosphere.

The amount of carbon in the samples was determined by thermogravimetric analysis (TGA; TA Instruments Q5000IR) in air by heating the powders from ambient temperature to 700 °C using a heating ramp of 10 °C min^−1^.

Electron paramagnetic resonance (EPR) spectroscopy was performed at room temperature on a Bruker MicroEMX spectrometer equipped with a Bruker super high Q resonator with a microwave frequency of 9.87 GHz, microwave power of 1 mW, field modulation of 100 kHz and modulation amplitude of 4 G. The field calibration was carried out using 2,2‐diphenyl‐1‐picrylhydrazyl (DPPH) as a standard and measurements were normalised to the sample weight.

X‐ray photoelectron spectroscopy (XPS) was carried out using a PHI 5500 XPS instrument with an Al Kα X‐ray source (1486.6 eV). Powder samples were mixed with a small amount of carbon black, using a mortar and pestle, to provide good electronic conductivity for the measurements. The energy was calibrated to the graphitic carbon (C=C) peak in the C 1s spectra (284.0 eV) for each sample. Data were analysed with the CasaXPS package software, employing the Gaussian‐Lorentzian peak shape GL(30).

^23^Na magic‐angle spinning (MAS) NMR spectra were acquired using a 700 MHz Bruker Avance III HD WB spectrometer at a magnetic field of 16.4 T. Experiments were performed using a Bruker 3.2 mm probe at a MAS rate of 10 kHz. Spectra were referenced relative to 1 m NaCl_(aq)_ solution using the ^23^Na resonance of solid NaCl at 7.5 ppm as a secondary reference.

UV‐visible spectroscopy was performed with a Cary500 spectrometer in the 200–500 nm range using an integrating sphere to acquire only diffusive reflectance of the electromagnetic radiation.

**Electrochemical characterisation**: The electrochemical performance of the sodium titanate samples synthesised in this work was tested using stainless steel CR2032 coin cells, Na metal as the counter/reference electrode (Alfa Aesar Merck), 1 m NaPF_6_ (99 % Alfa Aesar) in ethylene carbonate (EC): diethyl carbonate (DEC) solvent (battery grade, Gotion) (1:1 *v*/*v*) as the electrolyte and a Whatman micro glass fibre separator. The liquid organic electrolyte was dried for several days using activated molecular sieves (0.4 nm pore diameter, Merck) before use. The assembly and electrode preparation were carried out in an Ar filled glovebox (MBraun, H_2_O and O_2_ <0.1 ppm). The electrode preparation involved mixing of the active material (sodium titanate samples) with carbon black (Super P) (99 % Alfa Aesar) and polyvinylidene binder (PVDF Kynar, 99 % Alfa Aesar) in a weight ratio of 70:20:10, respectively. Electrode slurries were prepared by adding a few drops of *N‐*methyl‐2‐pyrrolidone (NMP) (anhydrous, 99 % Alfa Aesar) to the electrode mixture, which was stirred for 12 h. The obtained slurry was coated uniformly onto an aluminium foil using a doctor blade to form a film of 200 μm thickness which was then dried at 80 °C under vacuum for 12 h in the antechamber of the glovebox. Subsequently, the electrodes were cut into circular disks of 19 mm diameter with a load of active material of ca. 1.25 mg cm^−2^.

Galvanostatic charge/discharge measurements were performed on a battery tester (Neware battery system, current range: 1–10 mA) in the voltage range 0.01–2.5 V vs. Na^+^/Na at different rates (0.1, 0.2, 1 and 2 C). Cyclic voltammetry measurements were conducted on an Ivium potentiostat (Alvatek) in the voltage range 0.01–2.5 V vs. Na^+^/Na at different scan rates (0.05, 0.1, 0.2 and 0.3 mV s^−1^). Mott‐Schottky measurements were carried out on an Ivium potentiostat (Alvatek), in the voltage range 0.01–2.5 V vs. Na^+^/Na using a frequency of 500 Hz with a scan step of 50 mV. Electrochemical impedance spectroscopy (EIS) data were collected on an Ivium potentiostat (Alvatek) with an AC amplitude of 10 mV in the frequency range between 0.05 and 10^5^ Hz. Data were acquired during the first discharge process at OCV (≈2.5 V), 1, 0.4, 0.2 and 0.01 V vs. Na^+^/Na.

**Ab initio calculations**: All the calculations performed in this work used the density functional theory (DFT) method as implemented in the Vienna Ab initio Simulation Package code.[^25^, ^26^] The projector augmented wave approach^[27]^ was employed to describe the interaction between the core and valence electrons. The electron configurations Na (3s^1^), Ti (3d^3^4s^1^), and O (2s^2^2p^4^) were treated as the valence electrons. Brillouin zones for all compounds were sampled such that the *k‐*points were converged in an accuracy of the total energy in 0.001 eV atom^−1^ (Table S1) and the plane‐wave cut‐off was set to be 500 eV to sufficiently converge the total energy to within 0.01 eV atom^−1^. In this relaxation, the atomic positions, lattice vector, and cell angle were allowed to relax. All calculations were deemed to be converged when the forces on all atoms were less than 0.01 eV Å^−1^.

The revised Perdew–Burke–Ernzerhof Generalised Gradient Approximation (GGA) functional (PBEsol)^[28]^ was used for all phase stability calculations including enthalpy and vibrational entropy. PBEsol has accurately reproduced lattice parameters and lattice dynamics in solid systems while maintaining a relatively low computational cost.[^29^, ^30^] The enthalpy (H ) was calculated for 20 different phases of the Na‐Ti‐O system obtained from the Materials Project database (Table S1).^[31]^ The vibrational entropy ($S_{\mathrm{vib}}$ ) was calculated using the supercell and finite displacement approaches, as implemented in the Phonopy package (Figure S1).^[32a]^ For the entropy calculations, all the structures were initially relaxed to a force convergence criterion of 0.0001 eV Å^−1^. The harmonic force constants and associated vibrational entropy were then calculated by creating atomic displacements in 4×4×4 (128 atoms), 1×2×3 (36 atoms), 3×3×3 (324 atoms), 2×3×2 (288 atoms), 1×3×2 (252 atoms), 1×3×2 (252 atoms), 3×2×2 (216 atoms), 2×1×1 (212 atoms), 1×4×2 (192 atoms) supercells of Na, Ti, Na_2_O, Na_2_Ti_3_O_7_, Na_2_Ti_6_O_13_, Na_4_Ti_5_O_12_, Na_4_TiO_4_, Na_8_Ti_5_O_14_, and TiO_2_, respectively. In this calculation, O_2_ gas was modelled by placing a molecule in a cubic box of 30 Å in length. The Gibbs free energies (*G* ${}_{<\mathrm{Na}{}_{x}\mathrm{Ti}{}_{y}O{}_{z}}$ ) of seven different phases (Na_2_O, Na_4_TiO_4_, Na_8_Ti_5_O_14_, Na_4_Ti_5_O_12_, Na_2_Ti_3_O_7_, Na_2_Ti_6_O_13_, and TiO_2_) lying on a tie line between Na_2_O and TiO_2_ were calculated as [Eq. 1]:

(1) $$G_{Na_{x}Ti_{y}O_{z}\;}=\;H-TS_{\mathrm{vib}}$$

where *T* is the absolute temperature. The free energies of formation for Na_*x*_Ti_*y*_O_*z*_ (Δ*G* ${}_{<f,\mathrm{Na}{}_{x}\mathrm{Ti}{}_{y}O{}_{z}}$ ) can then be calculated as [Eq. 2]:

(2) $$\Delta G_{f,\;Na_{x}Ti_{y}O_{z}}\;=\;G_{Na_{x}Ti_{y}O_{z}}-xG_{\mathrm{Na}}-yG_{\mathrm{Ti}}-\frac{z}{2}G_{O_{2}}$$

where *G*_Na_, *G*_Ti_, and *G* ${}_{O{}_{2}}$ are the free energies of bulk Na, Ti, and O_2_ gas.

All electronic structure and band alignment calculations were performed using the screened hybrid functional (HSE06),^[33]^ in which 25 % of exact non‐local Fock exchange is added to the PBE^[34]^ functional. The structures of bulk unit cells of Na_2_Ti_3_O_7_ and Na_2_Ti_6_O_13_ were first optimised using the HSE06 functional. The electronic band structures and density of states were then generated from the optimised HSE06 bulk unit cell using the open source Python package: sumo developed by Ganose et al.^[32b]^ The band offsets between Na_2_Ti_3_O_7_ and Na_2_Ti_6_O_13_ were determined by aligning the valence band maximum (VBM) of each phase to the vacuum level.[^35^, ^36^] We first constructed the two different Na_2_Ti_3_O_7_ and Na_2_Ti_6_O_13_ slab structures by stacking four (100) layers of Na_2_Ti_3_O_7_ and Na_2_Ti_6_O_13_, followed by adding a vacuum layer with a thickness of 30 Å. The macroscopic planar‐averaged potentials were then evaluated to obtain electrostatic potentials (*E*_vac_) in the vacuum space. The electrostatic calculations showed that the constructed slabs were thick enough to allow the core state energy (*E*_core,slab_) of a Na atom located in the middle of the slab to represent that of bulk structure. Finally, the VBM level with respect to the vacuum level, that is, ionisation potential (*IP*), was calculated by comparing the obtained ($E_{\mathrm{vac}}$ ) and (*E*_core,slab_) 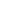 values with VBM (*E*_VBM,bulk_) and core levels (*E*_core,slab_) of bulk structures according to [Eq. 3]:

(3) $$\mathrm{IP}\;=\;E_{\mathrm{vac}}-\;E_{\mathrm{VBM},\mathrm{bulk}}\;=\;\left(E_{\mathrm{vac}}\;-{\;E}_{\mathrm{core},\mathrm{slab}}\right)\;-\;\left(E_{\mathrm{VBM},\mathrm{bulk}}\;-{\;E}_{\mathrm{core},\mathrm{bulk}}\right)$$

 

## Results and Discussion

### Structure and Characterisation

**Figure 1:**
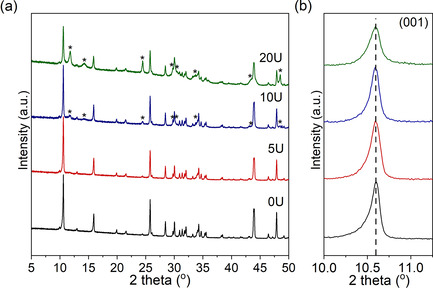
(a) PXRD data of 0U (black), 5U (red), 10U (blue) and 20U (green) samples at room temperature in the 5–50° 2*θ* range. Asterisk symbols correspond to diffraction peaks assigned to the Na_2_Ti_6_O_13_ phase (space group C2/m). (b) Zoom‐in of the 10–11.5 ° 2*θ* range in (a), showing the diffraction peak corresponding to (001) crystal plane.

**Figure 2:**
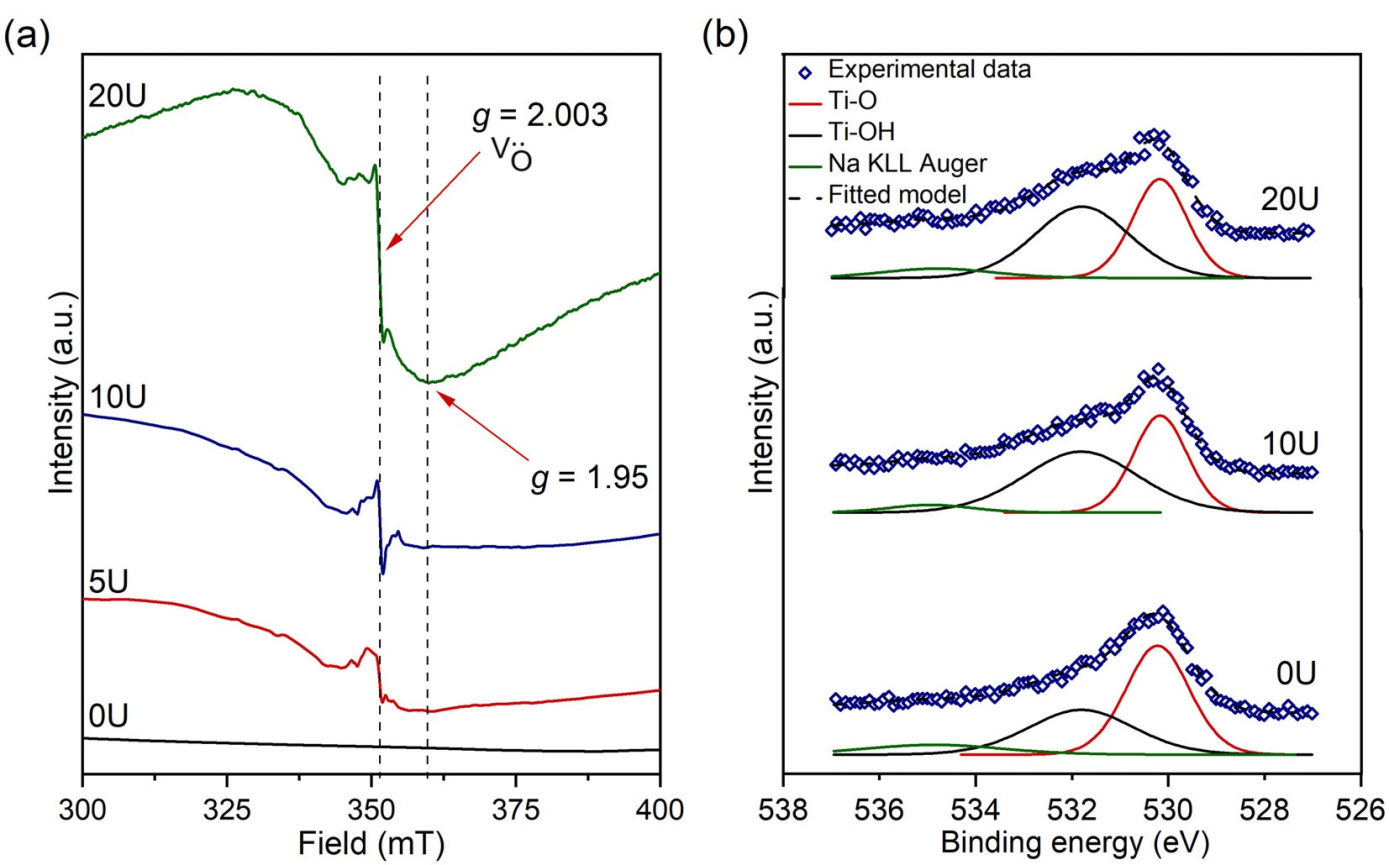
(a) EPR spectra in the magnetic field range 300–400 mT of samples 0–20U. (b) High‐resolution O 1s XPS spectra of samples 0U, 10U and 20U.

Figure 1 a shows the powder X‐ray diffraction (PXRD) data for pristine NTO (0U) and urea‐treated NTO samples (5–20U). Figure S2 shows the PXRD pattern of 0U (black) together with the calculated (red) and difference (blue) profiles obtained by Rietveld refinement. The resulting structural parameters are shown in Table S2. A good agreement is observed between the experimental data and the pattern calculated with NTO structural model (space group *P*12_1_/*m*1) reported in the literature.[^37^, ^38^] Furthermore, Figure 1 b shows that the most intense diffraction peak corresponding to the (001) crystal plane is present at identical 2*θ* values (i.e. 10.6°) in all the samples, indicating that the interlayer distance (*d*) does not change after the urea treatment (*d*≈8.57 Å). A secondary monoclinic phase with composition Na_2_Ti_6_O_13_ (space group *C*2/*m*, ICSD 23877) is observed in sample 10U and its content increases proportionally with the amount of urea used, implying that NTO undergoes partial decomposition during the urea treatment. Reports have shown that given the structural similarities between both sodium titanate phases, NTO may be converted to Na_2_Ti_6_O_13_ through sodium and oxygen loss upon heating at high temperature (950 °C) in air^[39]^ or at mild temperatures (400–500 °C) in a reducing atmosphere.[^10^, ^40^] Furthermore, some reports have shown the formation of Na_2_O in addition to Na_2_Ti_6_O_13_ during thermal decomposition of NTO.[^40^, ^41^, ^42^] Although PXRD data do not show evidence of Na_2_O, ^23^Na MAS NMR experiments evidenced the formation of Na_2_CO_3_ during the urea treatment, which might result from the reaction of Na_2_O with CO_2_ originated from the hydrolysis of fulminic acid, as will be discussed in detail later. Sodium titanate materials have been reported to exhibit Na and/or O non‐stoichiometry, while retaining their original crystal structure.[^43^, ^44^, ^45^] However, beyond a certain level of non‐stoichiometry, the structure of these materials become unstable and suffer partial decomposition. This could explain the formation of Na_2_Ti_6_O_13_ in samples 10–20U. A similar phenomenon was observed for LiV_3_O_8_ annealed at 450 °C in a reducing atmosphere (5 % H_2_/Ar).^[46]^ Despite the formation of a non‐stoichiometric sodium titanate phase in sample 5U (see EPR data, Figure 2 a) it was not possible to identify the Na_2_Ti_6_O_13_ phase in the PXRD pattern. This could be due to the low amount of urea mixed with the as‐synthesised NTO (5 wt %), which might yield a small amount of Na_2_Ti_6_O_13_ that is below the detection limit of the X‐ray diffractometer. When tested as a SIB anode, Na_2_Ti_6_O_13_ showed higher Na^+^ ion mobility than NTO due to its 3D tunnel structure but was no further considered for practical applications due to its low theoretical capacity (49.5 mAh g^−1^).[^47^, ^48^] Nevertheless, previous studies have shown that a suitable hybridisation of NTO and Na_2_Ti_6_O_13_ can improve the overall anode rate performance with respect to NTO alone.[^48^, ^49^] This might suggest that the formation of this phase could offset respective drawbacks stemming from its structure and enhance the overall Na‐storage behaviour. The formation of Na_2_Ti_6_O_13_ and its effect on the anode performance will be further discussed in the theoretical section.

Field emission electron microscopy (FESEM) images of the as‐prepared samples (Figure S3) show that their microstructure consist of elongated particles with several microns in length and some hundreds of nanometres in width, in agreement with literature reports on Na_2_Ti_3_O_7_.[^3^, ^11^] Despite retaining the morphology after the urea treatment, a broader particle size distribution is observed in those samples where a higher concentration of urea was used. Energy‐dispersive X‐ray analysis (EDX) shows a homogeneous elemental distribution of Na, Ti and O atoms across the particles in all the samples (Figure S4). Furthermore, combined thermogravimetric analysis (TGA, Figure S5) and transmission electron microscopy (TEM, Figure S6) suggest that the samples contain ≈9 wt. % of carbon (formed from sucrose pyrolysis), which is found as a uniform coating layer with a thickness of 15–17 nm on the surface of the sodium titanate particles.

Electron paramagnetic resonance (EPR) spectroscopy was used to unambiguously detect the presence of unpaired electrons trapped in the structure, including the formation of oxygen vacancy sites.^[50]^ Figure 2 a shows the EPR spectra of samples 0–20U. The EPR signal at 350 mT (*g=*2.003) is attributed to the presence of oxygen vacancies in NTO, as reported in the literature.[^9^, ^51^] Furthermore, a broad signal with a minimum at 360 mT (*g=*1.95) may be explained with the presence of paramagnetic Ti^3+^ ions.[^52^, ^53^] The calculations used to obtain both *g‐*values can be found in the Supporting Information (Eq. S1). From these data, it is observed that more oxygen vacancies and Ti^3+^ ions are generated with increasing urea content, as reflected by the increase in the intensity of the corresponding resonance peaks.

The surface chemical states of the samples were investigated by X‐ray photoelectron spectroscopy (XPS), as shown in Figures 2 b and S7. The high‐resolution Ti 2p XPS spectra (Figure S7 b) show peak doublets at 459.5 eV and 464.5 eV, which correspond to the binding energies of the Ti 2p_3/2_ and 2p_1/2_ peaks of Ti^4+^ ions, respectively.^[54]^ No peaks related to the presence of Ti^3+^ ions are observed in these data. This suggests that the concentration of Ti^3+^ ions on the surface of the urea‐treated samples is very diluted. These data are in agreement with previous XPS reported data on hydrogenated NTO samples, where an almost undetectable concentration of Ti^3+^ ions was observed.[^9^, ^10^] The high‐resolution O 1s XPS spectra (Figure 2 b) show a peak centred at 530.2 eV (red) which is attributed to Ti−O bonds, whereas the broader and less intense peak centred at 531.7 eV (black) is attributed to Ti−OH bonds.[^10^, ^54^] Ti−O and Ti−OH peaks in sample 20U decrease and increase in intensity, respectively, compared to the pristine material (0U), suggesting that more oxygen vacancies and hydroxyl groups are generated after the urea treatment.^[10]^ Furthermore, XPS analysis does not show any evidence of nitrogen present in the samples as a result of the thermal decomposition of urea and/or the direct reaction between the samples and the N_2_ atmosphere used during the heating treatment (Figure S7 a). Therefore, it is possible to rule out the formation of a N*‐*modified NTO compound.

^23^Na MAS NMR was performed on all the samples (Figures S8 and S9) to further probe their local structure. The presence of Ti^3+^ species would be expected to reduce the ^23^Na spin‐lattice relaxation time (*T*_1_) due to paramagnetic relaxation.[^55^, ^56^] The ^23^Na MAS NMR spectrum of NTO (Figure S8 a) exhibits two main signals centred at 3.5 ppm and −12 ppm, which correspond to the Na(1) (coordination number=7) and Na(2) (coordination number=9) ions, respectively.[^57^, ^58^] Samples 10U and 20U show peaks centred at 6.3 ppm and −18.6 ppm which increase with urea content and correspond to Na_2_CO_3_ and Na_2_Ti_6_O_13_, respectively (Figure S8 b).[^59^, ^60^] A possible mechanism for the formation of Na_2_CO_3_ is proposed later. As observed in Figure S8 a, the Na1 and Na2 line shapes do not change across the different samples. This indicates that defects might be formed either on the surface or at a bulk concentration which is too low to promote any global changes in the local Na coordination environments. Due to the overlap of the Na_2_CO_3_ and Na_2_Ti_6_O_3_ with the Na_2_Ti_3_O_7_ resonances, it was not possible to accurately measure the T_1_ relaxation time. However, T_1_ was estimated by integrating over a small region of the Na1 resonance. From samples 0–20U, the estimated T_1_ relaxation time decreased slightly from 5.6 to 4.8 s (Figure S9). This decrease could reflect the presence of Ti^3+^ defects although the small size of the reduction suggests that the concentration of the defects must be very low.

In conclusion, based on our experimental evidence, we propose that upon heating the NTO and urea mixture, the latter decomposes into ammonia (NH_3_) and fulminic acid (HCNO) at *T* ≈180 °C ([Eq. 4]),^[21]^ and then ammonia further decomposes into reactive H_2_ and inert N_2_ at *T*>400 °C ([Eq. 5]):^[21]^

(4) $$\mathrm{CH}{}_{4}N{}_{2}O{}_{\left(s\right)}\to \mathrm{NH}{}_{3(g)}+\mathrm{HCNO}{}_{\left(g\right)}\;\;\left(T\approx 180\;{}^{\circ }C\right)$$

(5) $$2\;\mathrm{NH}{}_{3(g)}\to N{}_{2(g)}+3\;H{}_{2(g)}\;\;\left(T>400\;{}^{\circ }C\right)$$

The reducing H_2_ gas then removes oxygen atoms from NTO creating anionic vacancies in the structure, as observed with EPR and XPS (Figure 2). The process can be regarded as [Eq. 6]^[61]^

(6) $$2{\;O}^{2-}\to \;O_{2}\;+\;4{\;e}^{-}$$

Upon oxygen loss, H_2_ combines with O_2_ to form H_2_O vapour^[62]^ and the liberated electrons are transferred to the empty 3d levels at the bottom of the conduction band belonging to the adjacent Ti atoms.^[63]^ Consequently, Ti^4+^ ions are reduced to Ti^3+^ ions (as seen in the EPR data (Figure 2 a)), generating unpaired electrons in the 3d shell of the Ti atom. The overall reaction process may be seen as [Eq. 7]:[^62^, ^64^]

(7) H2(g)+Na2Ti3O7(s)→H2O(g)+Na2Ti4+3-xTi3+xO7-y(s)

Furthermore, PXRD and ^23^Na MAS NMR data (Figure 1 and Figure S8) show the formation of the Na_2_Ti_6_O_7_ secondary phase, which is consistent with the creation of defects in the NTO structure.[^10^, ^40^] In addition, the ^23^Na MAS NMR data show the formation of Na_2_CO_3_ (Figure S8), which may be explained by two different reaction mechanisms; HCNO is reported to decompose to NH_3_ and CO_2_ when H_2_O is present in the medium through a hydrolysis process [Eq. 8]:[^64^, ^65^]

(8) $$\mathrm{HCNO}{}_{\left(g\right)}+H{}_{2}O\to \mathrm{NH}{}_{3(g)}+\mathrm{CO}{}_{2(g)}$$

Since H_2_O is formed upon reduction of NTO (Eq. 7), it is likely that this reaction (Eq. 8) occurs. The generation of CO_2_ could result in the formation of Na_2_CO_3_, either by the reaction of Na_2_O (formed upon decomposition of NTO) with CO_2_, or through an exchange of Na^+^ in NTO with H^+^ ions from H_2_O.^[57]^ During the process, Na^+^ reacts with the OH^−^ group of H_2_O, which in turn further reacts with CO_2_ to form Na_2_CO_3_.^[57]^

### Electrochemical testing in Na‐ion batteries

Electrochemical tests including galvanostatic charge/discharge (GCD) cycling, cycling voltammetry (CV) and electrochemical impedance spectroscopy (EIS) measurements were carried out to investigate the electrochemical properties of the as‐prepared electrodes. Figure S10 shows the galvanostatic charge/discharge voltage profiles of samples 0–20U in the voltage range 0.01–2.5 V vs. Na^+^/Na during cycles 1 and 2 at 0.1 C (17.7 mA g^−1^) cycling rate. Almost identical load curves were observed for all the samples, except for an additional slope at 0.8–0.6 V vs. Na^+^/Na (marked with an arrow in Figure S10) in sample 20U, attributed to the insertion of Na^+^ ions into the Na_2_Ti_6_O_13_ impurity, observed with PXRD and ^23^Na NMR (Figure 1 and Figure S8).[^49^, ^66^] The absence of this slope in the load curves of sample 10U may be attributed to the lower content of Na_2_Ti_6_O_13_ found in this sample.

During the first discharge process, the plateau at 0.6 V vs. Na^+^/Na corresponds to the irreversible reaction of Na^+^ ions with the carbon additive.[^6^, ^11^] Furthermore, the two plateaux at the voltage below 0.2 V vs. Na^+^/Na are related to two‐phase transitions corresponding to Na_2_Ti_3_O_7_→Na_3−*x*_Ti_3_O_7_ and Na_3−*x*_Ti_3_O_7_→Na_4_Ti_3_O_7_ reactions,^[7]^ which correspond to the insertion of two Na^+^ ions with concomitant reduction of Ti^4+^ to Ti^3+^ (C_theoretical_=177 mAh g^−1^).[^6^, ^11^] During the first charge process, a single plateau at 0.4 V vs. Na^+^/Na is observed, which corresponds to the extraction of Na^+^ from the Na_4_Ti_3_O_7_ structure.[^6^, ^11^] From the second cycle onwards, only the plateau at 0.2 V vs. Na^+^/Na is observed upon discharge, suggesting that the Na_2_Ti_3_O_7_→Na_3−*x*_Ti_3_O_7_ pathway process no longer occurs and, hence, Na_2_Ti_3_O_7_ transforms directly to Na_4_Ti_3_O_7_.^7^ Both NTO and urea‐treated samples show a very similar first discharge and charge capacities of ca. 420 mAh g^−1^ and 260 mAh g^−1^, respectively, with a low coulombic efficiency of ca. 60 % (Figure S10). The poor coulombic efficiency observed in the first cycle is explained by the irreversible reaction of Na^+^ ions with carbon (ca. 0.6 V vs. Na^+^/Na)[^6^, ^11^] and the formation of an SEI layer, which starts to form at voltages below 1.0 V vs. Na^+^/Na.[^67^, ^68^] In subsequent cycles, the coulombic efficiency increases to more than 98 % and remains constant up to cycle 100. The long‐term cycling performance of the 0–20U electrodes at the 0.1 C cycling rate (Figure S11) suggests a similar degradation pathway over 100 cycles. Furthermore, it should be noted that the presence of Na_2_CO_3_ (Figure S8) does not seem to have a detrimental effect on the overall electrochemical stability of samples 10U and 20U. These results are in accordance with the data reported by Tsiamtsouri et al., who showed that Na_2_CO_3_ did not have an effect on the cycling stability.^[58]^

**Figure 3:**
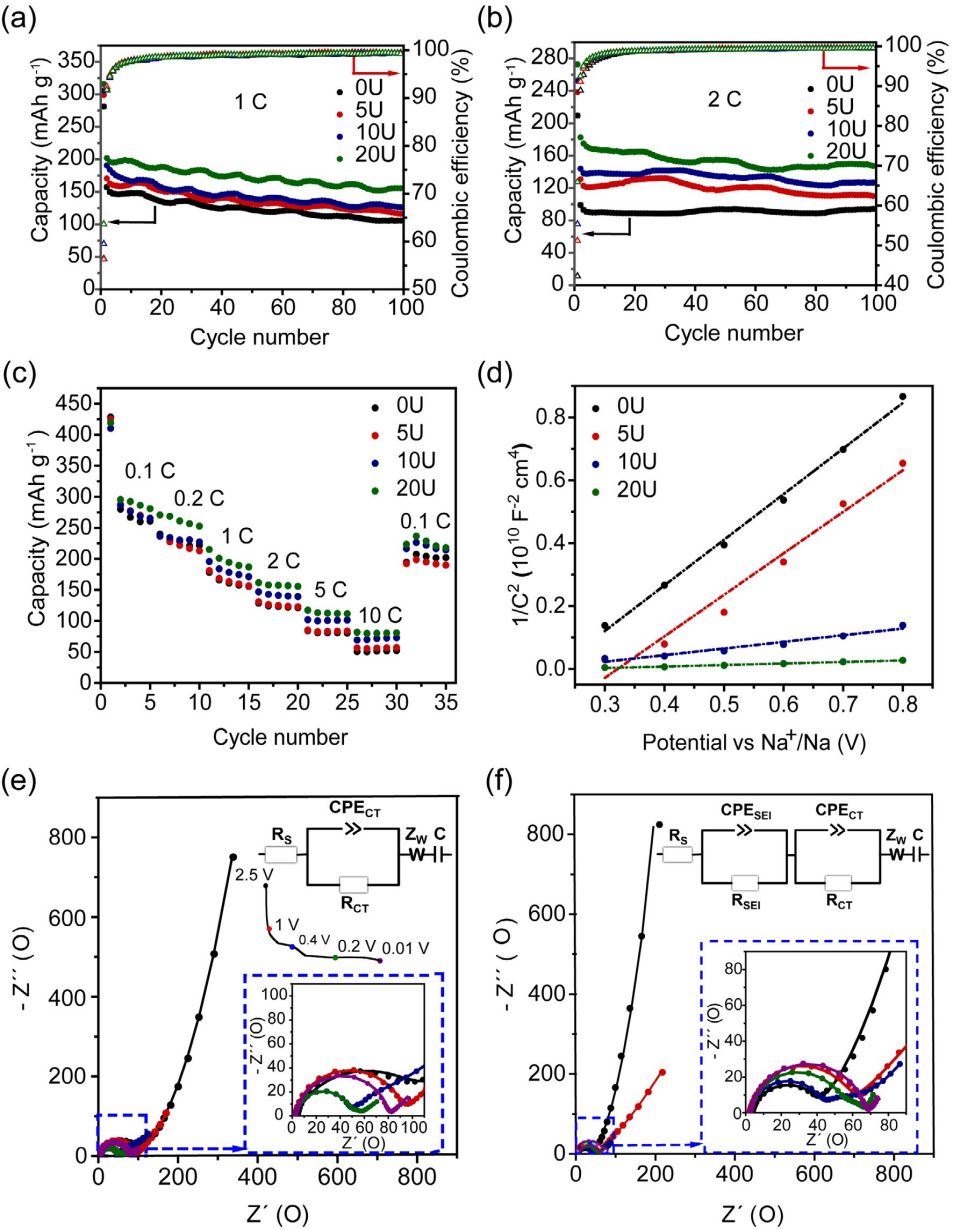
Long‐term cycling performance of samples 0–20U in the voltage range 0.01–2.5 V vs. Na^+^/Na over 100 cycles at (a) 1 C, (b) 2 C cycling rates; (c) Rate performance at different rates (0.1–10 C). Five charge/discharge cycles were run at each current rate; (d) Mott–Schottky plots of 0–20U samples at 500 Hz between 0.3 and 0.8 V vs. Na^+^/Na; Nyquist plots obtained at different states of charge during the first discharge cycle, from 100 MHz to 50 mHz for (e) 0U and (f) 20U electrodes.

Overall, a capacity retention upon charge of ca. 40 % was observed after 100 cycles. This could be the result of the formation of an unstable SEI layer and the continuous increase of the charge‐transfer resistance, commonly observed in previous studies.[^17^, ^69^, ^70^] These data suggest that the generation of defects in the crystal structure and the presence of Na_2_Ti_6_O_13_ do not lead to an improvement in the electrochemical performance at relatively low cycling rates. By contrast, galvanostatic cycling at 1 C (177 mA g^−1^) and 2 C (354 mA g^−1^) cycling rates (Figure 3 a,b) show a major improvement of the capacity with increasing content of urea. Thus, the 20U sample exhibits the best electrochemical performance with an initial discharge capacity of 316 mAh g^−1^ (1 C) and 272 mAh g^−1^ (2 C) (cf. 281 (1 C) and 210 mAh g^−1^ (2 C) for 0U). 5U and 10U show initial capacities of 299 (1 C), 238 mAh g^−1^ (2 C) and 306 (1 C), 252 mAh g^−1^ (2 C), respectively. By the end of cycle 100, 20U shows a discharge capacity of 154 mAh g^−1^ (1 C) and 145 mAh g^−1^ (2 C), in contrast to 106 mAh g^−1^ (1 C) and 90 mAh g^−1^ (2 C) for 0U.

Figure 3 c shows the rate performance of the samples at different cycling rates. At all rates, the 20U performs better than the other samples. At the high rates of 2, 5 and 10 C, the discharge capacities are 160, 112 and 80 mAh g^−1^, respectively. Once the rate decreases from 10 C to 0.1 C, the discharge capacity of all the electrodes increases significantly, reaching approximately 80 % of their initial capacity. The capacity is kept fairly constant in the following five cycles, suggesting good structural stability and reversibility.[^9^, ^16^] In conclusion, the galvanostatic data show that the urea treatment significantly increases the rate capability and specific capacities of the active materials, leading to enhanced electrochemical performance. The improvement is due to a synergistic combination of defects generated in NTO (oxygen vacancies, Ti^3+^ and Ti‐OH species) and the formation of Na_2_Ti_6_O_13_ which enable high Na^+^ ion/e^−^ mobility. These results outperform many of the NTO systems reported in the literature (Table S3).

To gain further insight into the electrochemical behaviour of the different materials studied, CV and EIS measurements were conducted. Figure S12 shows the CV curves for samples 0–20U at various scan rates from 0.05 to 0.3 mV s^−1^ in the voltage range 0.01–2.5 V vs. Na^+^/Na. In a typical CV for NTO at a low scanning rate, the first cathodic sweep involves the reaction of Na^+^ ions with the carbon additive at 0.36 V vs. Na^+^/Na and the insertion of Na^+^ into the layered NTO structure at 0.08 V vs. Na^+^/Na, concomitant with the reduction of Ti^4+^ to Ti^3+^ ions.[^11^, ^71^] Moreover, electrolyte reduction starts to take place at voltages below 1.0 V vs. Na^+^/Na and becomes more severe at deep discharge voltages (0.01 V).[^67^, ^68^] Upon charge, only the anodic peak related to the extraction of Na^+^ ions from the NTO structure is observed.[^11^, ^71^] As the scanning rate increases, the anodic and cathodic peaks shift towards lower and higher voltages, respectively, suggesting that the insertion/extraction of Na^+^ ions becomes more sluggish.^[54]^ Furthermore, the oxidation current peaks are higher than the reduction peaks, indicating that the extraction process of Na^+^ ions is more facile than the insertion process.

In a diffusion‐controlled process, the peak current is proportional to the square‐root of the scanning rate.^[72]^ Figure S13 shows a linear relationship between these two parameters, which confirms the intercalation process in all the samples to be diffusion‐controlled. Furthermore, the diffusion coefficient of Na^+^ ions, $D_{{\mathrm{Na}}^{+}}$ , can be determined using the Randles–Sevcik relationship (Eq. S2). The calculated intercalation $D_{{\mathrm{Na}}^{+}}$ increases with increasing defect content (i.e. 0U (1.2×10^−11^ cm^2^ s^−1^), 5U (1.7×10^−11^ cm^2^ s^−1^), 10U (2.7×10^−11^ cm^2^ s^−1^) and 20U (4.1×10^−11^ cm^2^ s^−1^)). Mott‐Schottky plots obtained from EIS recorded at a frequency of 500 Hz (Figure 3 d) provide evidence of higher charge carrier density due to the increased defects induced by the urea treatment. These are calculated to be 6.2×10^22^ cm^−3^ (0U), 1.09×10^23^ cm^−3^ (5U), 4.14×10^23^ cm^−3^ (10U) and 2.02×10^24^ cm^−3^ (20U) using Eq. S3. Furthermore, EIS data were collected during the first discharge process at different states of charge (open circuit voltage (OCV≈2.5 V), 1.0, 0.4, 0.2 and 0.01 V vs. Na^+^/Na) at the 0.1 C cycling rate. The Nyquist plots derived from the EIS measurements on samples 0U and 20U in the frequency range from 100 MHz to 50 mHz are shown in Figures 3 e,f. The obtained spectra in the OCV state were fitted using the equivalent circuit shown in Figure 3 e (inset), which is composed of a *R*_s_ at high frequencies, a charge‐transfer resistance (*R*_CT_) along with a constant phase element (*CPE*), a Warburg impedance (*Z*_W_) and a capacitance (*C)*. The intercept at the Z’ axis in the high‐frequency range (*R*_s_) is dominated by the resistance of the electrolyte to ion transport; the high‐medium frequency semicircle (*R*_CT_) refers to the charge‐transfer resistance for electrons and Na^+^ ions across the electrode‐electrolyte interface;^[73]^ the *CPE* corresponds to the electrical double layer capacitor on the interface between the electrode and electrolyte; and the slope observed in the low‐frequency domain is attributed to the Warburg impedance (*Z*_W_) and corresponds to the Na^+^ ion diffusion in the bulk of the electrode, followed by the chemical capacitance of the electrode (*C*) at very low frequencies.^[73]^ The estimated charge‐transfer resistances (*R*_CT_) are shown in Table S4. In the OCV state, the *R*_CT_ of 20U is 40.0 Ω, much lower compared to 0U (i.e. 104.2 Ω). This suggests that the charge transfer process is enhanced when defects are generated in the sodium titanate structure, in accordance to the enhanced electrochemical performance observed at high cycling rates (Figure 3 a–c). During discharge, the electrolyte starts to decompose at voltages below 1.0 V vs. Na^+^/Na,[^67^, ^68^] leading to the formation of an SEI layer on the surface of the electrode, as observed in the CV measurements (Figure S12). As the voltage further decreases, the decomposition continues to take place, leading to the formation of a thicker and more resistive SEI layer (Table S4).^[69]^ As a result, an additional *R*_SEI_ *CPE*_SEI_ in series with *R*_CT_ *CPE*_CT_ was added to the equivalent circuit as depicted in Figure 3 f. When the voltage decreases from 1.0 to 0.4 V vs. Na^+^/Na, the semicircle progressively contracts, showing charge‐transfer resistances of 28.4 Ω and 19.1 Ω for 0U and 20U, respectively. At this voltage, the SEI layer formation is clear from the presence of an additional semicircle at high frequencies. The impedance recorded at 0.2 V vs. Na^+^/Na shows that the charge‐transfer resistance is 18.1 Ω and 13.1 Ω for 0U and 20U, respectively. An increase of the SEI layer resistance is observed, confirming that the reduction of the electrolyte becomes more severe as the voltage decreases. These results demonstrate an improved charge transfer process occurring in the urea‐treated samples which may explain their enhanced rate capability (Figures 3 a,c).

### Ab initio calculations

To further understand the origin of the enhanced electrochemical rate performance of samples 5–20U, we carried out two different computational analyses which correspond to the determination of the phase stability of NTO under urea treatment and the calculation of the band structures of NTO and Na_2_Ti_6_O_13_ and their energetic alignments.

**Figure 4:**
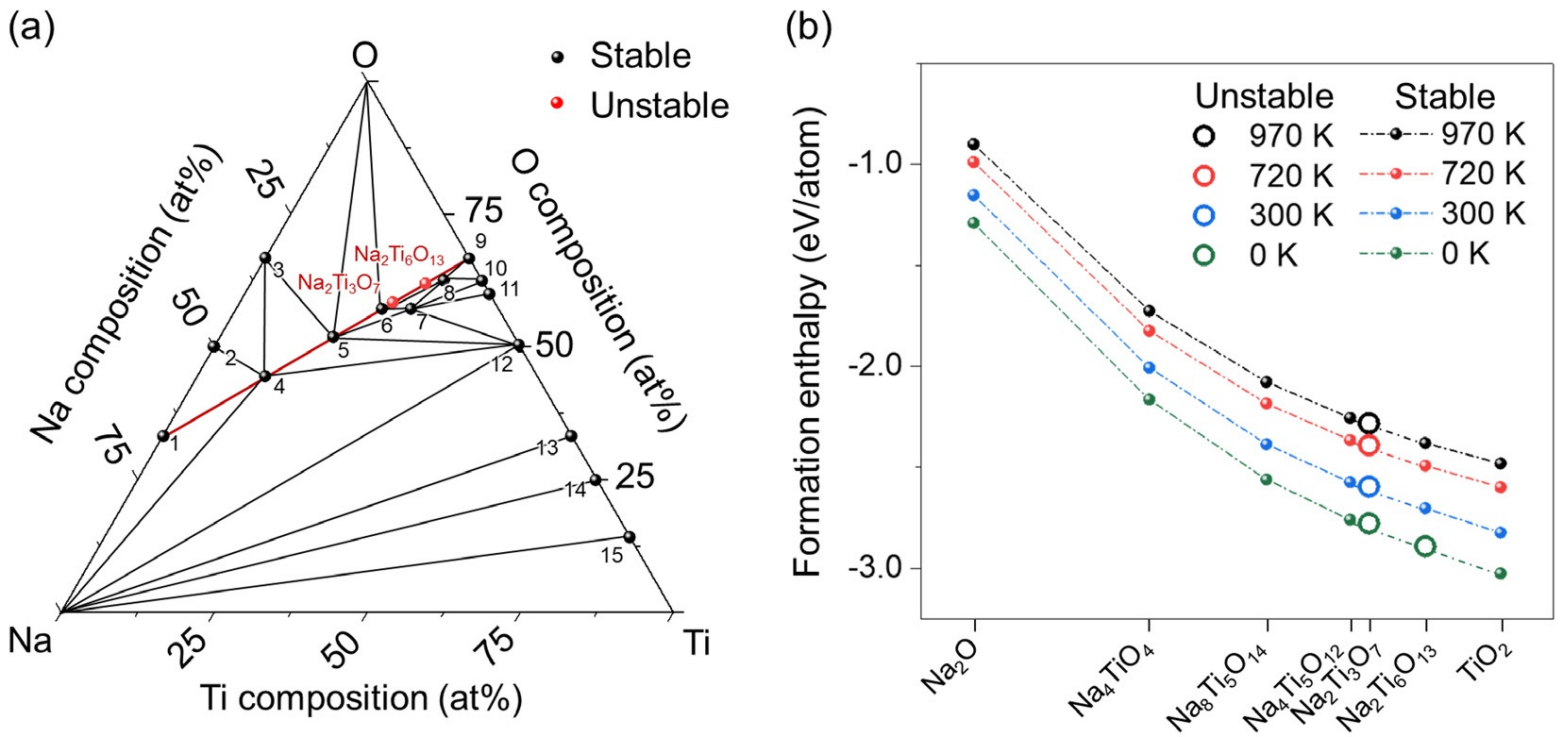
(a) Na‐Ti‐O phase diagram defined by Na, Ti and O obtained from structures with the lowest formation enthalpy at 0 K. Thermodynamically stable and unstable structures are indicated by black and red circles, respectively. The tie line including NTO and Na_2_Ti_6_O_13_ is shown in red. The numbers in (a) indicate the phases corresponding to: 1: Na_2_; 2: Na_2_O_2_; 3: NaO_2_; 4: Na_4_TiO_4_; 5: Na_8_Ti_5_O_14_; 6: Na_4_Ti_5_O_12_; 7: NaTi_2_O_4_; 8: NaTi_5_O_10_; 9: TiO_2_; 10: Ti_3_O_5_; 11: Ti_2_O_3_; 12: TiO; 13: Ti_2_O; 14: Ti_3_O; 15: Ti_6_O. (b) Gibbs free energy of formation calculated for seven phases lying on a tie line between Na_2_O and TiO_2_.

The stability of NTO is first analysed by comparing its thermodynamic stability with those of other stable phases reported for the Na‐Ti‐O system. This is achieved by obtaining stable atomic configurations of NTO and 19 stable compositions of Na_*x*_Ti_*y*_O_*z*_,^[31]^ (Table S1) followed by the calculation of the formation enthalpies of the obtained phases (see Experimental Section). Figure 4 a shows the Na‐Ti‐O phase diagram at 0 K, constructed based on the PBEsol calculated formation enthalpies. The analysis in Figure 4 a illustrates that NTO is unstable and decomposes into phases on a tie line between Na_2_O and TiO_2_. The tie line includes Na_2_Ti_6_O_13_, which seemingly explains the formation of the Na_2_Ti_6_O_13_ secondary phase during the urea treatment (Figure 1 a). However, a close examination on the ternary phase diagram reveals that Na_2_Ti_6_O_13_ is also thermodynamically unstable and, hence, not likely to be formed upon the decomposition of NTO. This discrepancy between the calculations and experiments may arise from the difference in temperatures used in the calculations (0 K) and in the urea treatment (723 K).

To analyse the phase stability during the urea treatment, Gibbs free energy of formation is calculated at elevated temperatures (300, 720, and 970 K). Figure 4 b shows the Gibbs free energies of formation of seven phases on the tie line between Na_2_O and TiO_2_. As the temperature increases, calculations start to place the free energy of Na_2_Ti_6_O_13_ below the convex hull. This suggests that Na_2_Ti_6_O_13_ becomes thermodynamically stable at elevated temperature, allowing the decomposition of NTO into Na_2_Ti_6_O_13_ during the urea treatment. Further calculations reveal that, under urea treatment at 450 °C (=723 K), the decomposition of NTO can cause changes in the Gibbs free energy (Δ*G*) from −6.7 to 193.4 meV atom^−1^ (Figure S14). The calculated Δ*G* reveals that most decomposition processes where Na_2_Ti_6_O_13_ forms require energy smaller than the thermal energy imposed during the urea treatment, that is, k*T*=62.3 meV atom^−1^, where *k* is the Boltzmann constant and *T* is the absolute temperature (723 K). This further supports the formation of Na_2_Ti_6_O_13_ during the urea treatment as observed in the PXRD and ^23^Na NMR data (Figure 1 and Figure S8). However, the above thermodynamic analysis neglects atomic displacements and associated kinetic barriers that also play an important role in the actual decomposition processes. To consider the kinetic contributions, we compared the atomic configuration of NTO with the other phases of the Na‐Ti‐O system (Figure S15). The comparisons show that, among the various phases in the Na‐Ti‐O system, Na_2_Ti_6_O_13_ has the most similar atomic configurations compared to NTO.[^10^, ^39^, ^40^] Thus, it is the most favourable phase with the lowest kinetic barriers. The phase transition from NTO to Na_2_Ti_6_O_13_ is particularly more likely to occur for the experiments performed under N_2_/H_2_ atmosphere, because the reactive H_2_ gas tends to remove Na and O from NTO^40^ which, in turn, facilitates the atomic movements for the phase transition from NTO to Na_2_Ti_6_O_13._ ^[42]^ From this perspective, the urea treatment (Eq. (4)–(5)) causes NTO to partially decompose into the Na_2_Ti_6_O_13_ secondary phase.

**Figure 5:**
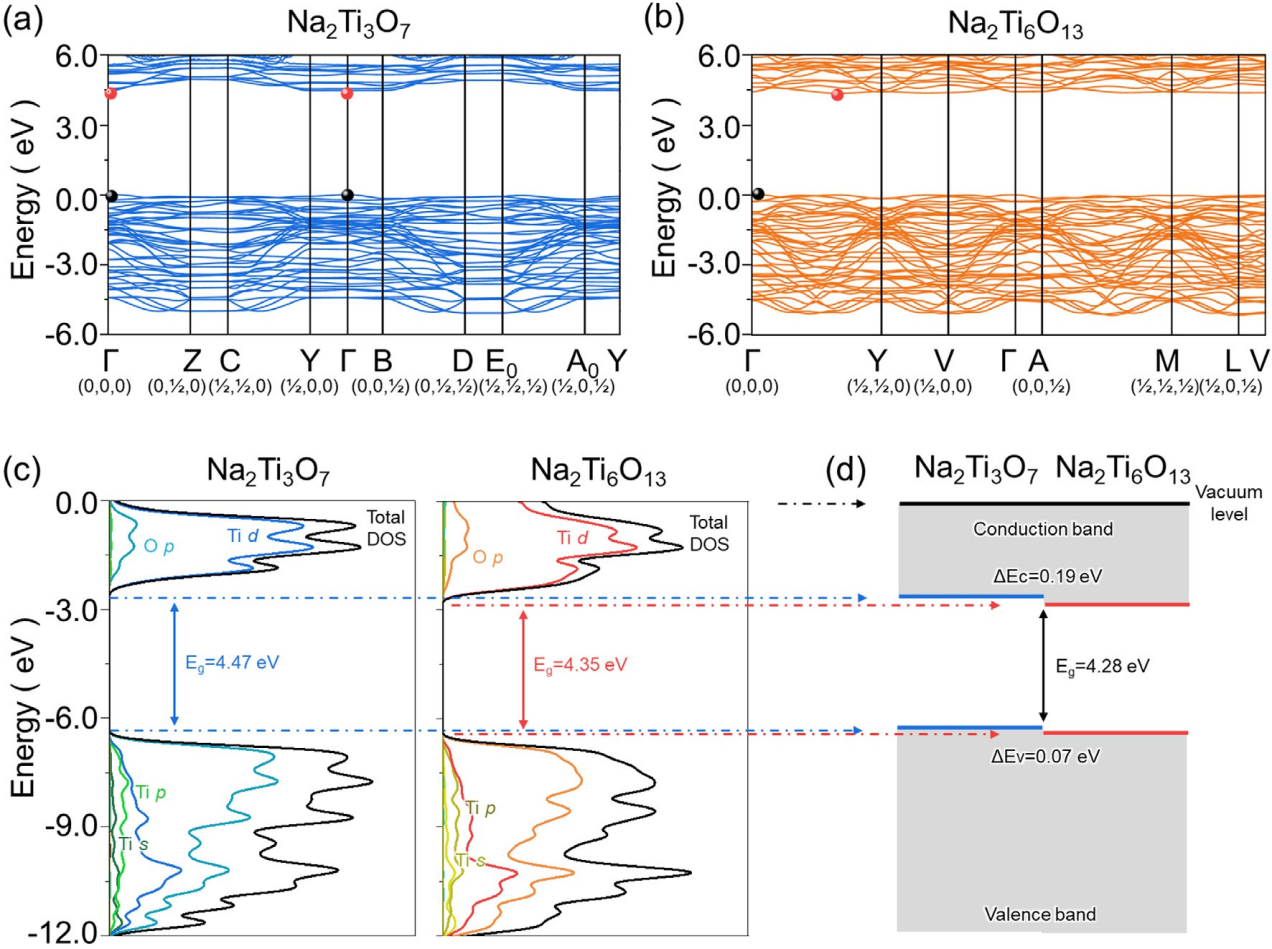
The electronic band structures of (a) NTO and (b) Na_2_Ti_6_O_13_ along with the high‐symmetry points in the Brillouin zone according to the Bradley and Cracknell notation.^[85]^ The valence band maximum (VBM) and conduction band minimum (CBM) are denoted by black and red circles, respectively. (c) Comparison of the total and ion‐decomposed electronic density of states of NTO and Na_2_Ti_6_O_13_ plotted with reference to the vacuum level at 0 eV. (d) Schematic of the band alignment of NTO and Na_2_Ti_6_O_13_.

Having identified Na_2_Ti_6_O_13_ as the most plausible phase formed during the urea treatment, we next focussed on the effect of Na_2_Ti_6_O_13_ on the electrochemical performance in samples 5–20U. One effective way to do this is to analyse the band structures of the constituents Na_2_Ti_3_O_7_ and Na_2_Ti_6_O_13_ that affect the electrical conductivity of anodes. Previous DFT works have calculated the band structure and associated electronic properties of NTO.[^9^, ^74^] However, all calculations to date were performed based on semi‐local exchange functionals such as GGA and LDAs, which largely underestimate the fundamental bandgaps,[^75^, ^76^] inhibiting an accurate assessment of the electronic properties of NTO. To effectively eliminate the bandgap underestimation error, we carried out band structure calculations for NTO and Na_2_Ti_6_O_13_ using an HSE06 hybrid functional that provides a more accurate description of the electronic structures of Ti‐containing materials (Figures 5 a,b).[^77^, ^78^, ^79^, ^80^, ^81^] Overall, the band structures of both materials are characterised by a wide bandgap with flat CBM, indicating that both phases are of electronic insulating nature. The flat CBM is more evident for NTO, ranging from the γ high symmetry point (Γ) to Y and B high symmetry points (Figure 5 a). A close examination of the CBM reveals that the direct transition of Γ→Γ in NTO has an energy just 1.9 and 6.6 meV smaller than the indirect gap transitions of Γ→Y and Γ→B. Such flat CBM can render NTO to undergo the direct‐indirect bandgap transition under minor changes in atomic structures, which makes the type of bandgap of NTO rather controversial, as usually reported in the literature.[^3^, ^74^, ^82^, ^83^] Another finding is that the fundamental bandgap of Na_2_Ti_6_O_13_ (4.35 eV) is relatively lower than that of NTO (4.47 eV), which could partially explain the decrease in the bandgap of NTO after the urea treatment, as observed by diffuse reflectance UV‐VIS spectroscopy (Figure S16), where a bandgap of 3.63 eV in the 20U sample was obtained compared to 3.88 eV for pristine NTO. In the case of a composite of NTO and Na_2_Ti_6_O_13_, the effective bandgap measured during the experiments can be further lowered, as the energetic alignment of the VBM and CBM in each phase also affects the size of the effective bandgap.^[77]^ To test the band alignment of NTO and Na_2_Ti_6_O_13_, we projected the band structures onto atomic orbitals of Na, Ti and O and then aligned them with respect to the vacuum level, as illustrated in Figures 5 c,d (see Experimental Section). Calculations predict the VBM and CBM of Na_2_Ti_6_O_13_ to be lower than NTO by 0.07 and 0.19 eV, respectively, causing an overall band structure of Na_2_Ti_6_O_13_ to lie below NTO. This alignment in band structures further decreases the effective bandgap at the interface such that the bandgap of the NTO composite anode is reduced by 0.19 eV, compared to NTO. The decrease in bandgap is in good agreement with the UV‐vis spectroscopy analysis (Figure S16), which showed a decrease of the bandgap by 0.25 eV after the urea treatment of NTO. Further examination of Figure 5 c confirms that the conduction band of both phases is mostly composed of Ti d orbital, which is consistent with previous literature.[^74^, ^84^] This indicates that, when O vacancies are generated in NTO structure, the resulting excess of electrons from the O vacancy will be centred on Ti cations. This can cause the reduction of Ti^4+^ to Ti^3+^ and, in turn, further decrease the bandgap of NTO anode. The decrease in bandgap could result in better electronic conductivity and, consequently, in an enhancement of the rate performance. In conclusion, the above calculations suggest that the enhanced anode performance of the urea treated samples is attributed to the synergetic effect of the Na_2_Ti_6_O_13_ secondary phase and O vacancy generated upon urea treatment.

## Conclusions

In summary, we have reported a safe, controllable and affordable method using urea at mild temperatures to synthesise a series of sodium titanate samples with different levels of oxygen vacancies. The formation of oxygen vacancies leads to the reduction of Ti^4+^ to Ti^3+^ ions in NTO, together with the formation of hydroxyl groups and a secondary phase, Na_2_Ti_6_O_13_. The urea‐treated samples showed superior electrochemical performance at high rates with respect to pristine NTO. The enhanced electrochemical performance agrees with the higher Na^+^ ion diffusion coefficient, higher charge carrier density and reduced bandgap observed in these samples, without the need of nanosizing and/or complex synthetic strategies. The optimal electrochemical performance among the series is found in sample 20U, which displays initial discharge capacities of 316 mAh g^−1^ (1 C) and 272 mAh g^−1^ (2 C) and discharge capacities of 154 mAh g^−1^ (1 C) and 145 mAh g^−1^ (2 C) which are significantly higher than those observed in the pristine material (106 mAh g^−1^ (1 C) and 90 mAh g^−1^ (2 C)) after 100 cycles. The creation of oxygen vacancies has shown to be a promising strategy to improve the performance of NTO electrodes for SIBs by circumventing their inherent low electronic conductivity. However, a complete understanding of the role of oxygen vacancies in the electrochemical performance of NTO is still very limited at this stage. There are still questions that remain unanswered due to their challenging quantification and spatial distribution. In addition, to fully comprehend the role of oxygen vacancies, other factors including the generation of Ti^3+^ and Ti‐OH groups and the formation of Na_2_Ti_6_O_13_ must be excluded. Similarly, it is equally important to understand the individual contribution of these components in the overall electrochemical performance of NTO. The method for enhancing the electrochemical performance demonstrated in this study could be extrapolated to other anode materials that suffer from poor Na^+^ ion/e^−^ kinetics, to potentially foster the practical application of advanced SIB anode materials beyond hard carbons.

## Conflict of interest

The authors declare no conflict of interest.

## Biographical Information

*Nuria Tapia‐Ruiz obtained her Ph.D. from the University of Glasgow (UK) in 2013. She then worked as a Research Fellow in the team of Prof. Bruce at the University of St. Andrews and the University of Oxford (UK) in 2013–2016. Currently, she is a Senior Lecturer in the Department of Chemistry at Lancaster University. Her research interests include the understanding of the structure‐property‐performance relationships in materials for energy storage such as monovalent and multivalent batteries and supercapacitors*.

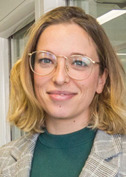

## Supporting information

As a service to our authors and readers, this journal provides supporting information supplied by the authors. Such materials are peer reviewed and may be re‐organized for online delivery, but are not copy‐edited or typeset. Technical support issues arising from supporting information (other than missing files) should be addressed to the authors.

Supplementary
