# Supplementary material for: Surface Engineering Strategy Using Urea To Improve the Rate Performance of Na2Ti3O7 in Na‐Ion Batteries
Source: Chemistry. 2021 Jan 14;27(11):3875–86. doi: 10.1002/chem.202003129 (PMC7986851; doi:10.1002/chem.202003129)
Supplement: Supplementary file 1 — Supplementary [file CHEM-27-3875-s001.pdf]

Chemistry–A European Journal

Supporting Information

Surface Engineering Strategy Using Urea To Improve the Rate Performance of $Na_2Ti_3O_7$ in Na-Ion Batteries

Sara I. R. Costa,[a, b] Yong-Seok Choi,[b, c, d] Alistair J. Fielding,[e] Andrew J. Naylor,[f] John M. Griffin,[a] Zdeněk Sofer,[g] David O. Scanlon,[b, c, d, h] and Nuria Tapia-Ruiz*[a, b]

Table of Contents

List of Figures……………………………………………………………………………………….2

List of Tables………………………………………………………………………………………18

List of Equations…………………………………………………………………………………..22

References………………………………………………………………………………………..25

List of Figures

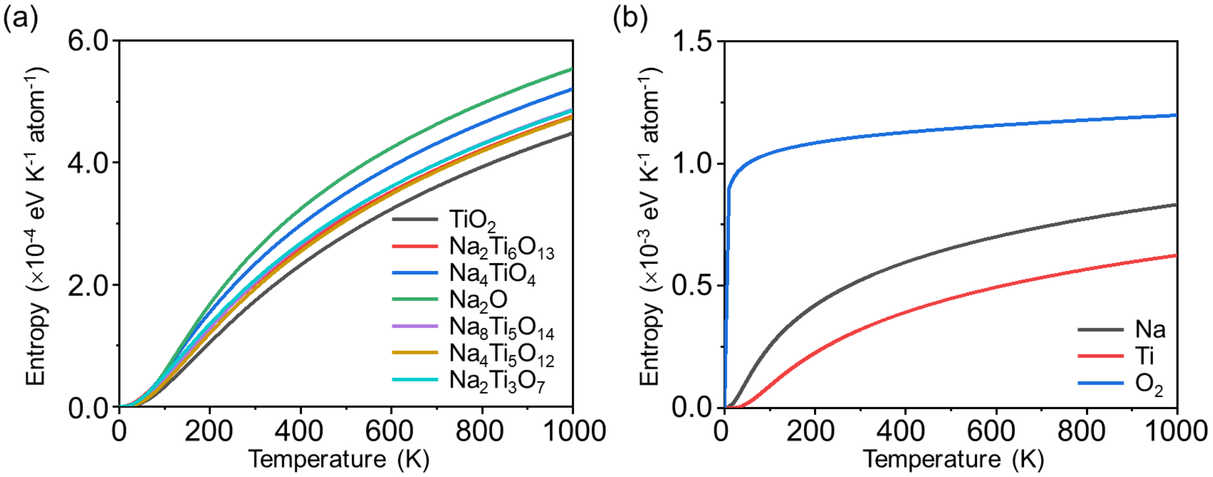

Figure S1. Vibrational entropies calculated for: (a) Na_2O, Na_4TiO_4, $Na_8Ti_5O_{14}$, $Na_4Ti_5O_{12}$, $Na_2Ti_3O_7$, $Na_2Ti_6O_{13}$ and TiO_2 and (b) the elemental phases of Na, Ti and O_2 plotted as a function of temperature.

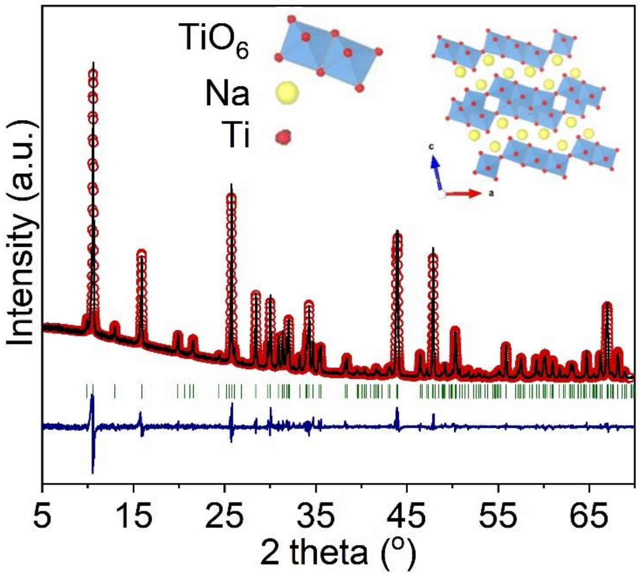

Figure S2. Rietveld fit of NTO (0U) using a $P12_1/m1$ structure model (ICSD 250000) against room temperature PXRD data. The black line corresponds to the observed data; the red circles indicate the calculated profile and the blue line is the difference between the two profiles. Green tick marks indicate Bragg reflections of $Na_2Ti_3O_7$. The inset shows a crystallographic representation of the NTO structure using VESTA software[1], where the blue polyhedra represent octahedrally coordinated Ti^{4+} ions, the yellow spheres are the Na^+ ions and the O^{2-} ions are represented in red.

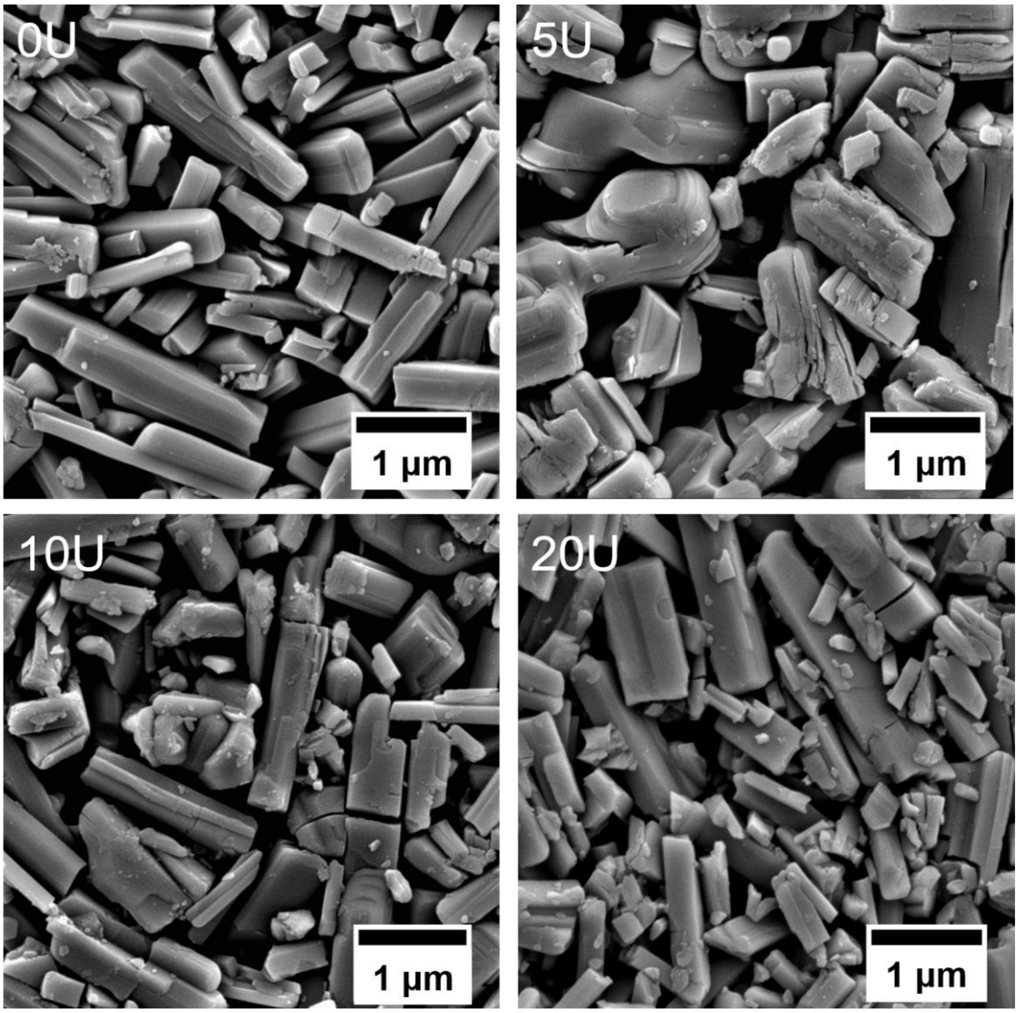

Figure S3. FESEM images of samples 0-20U.

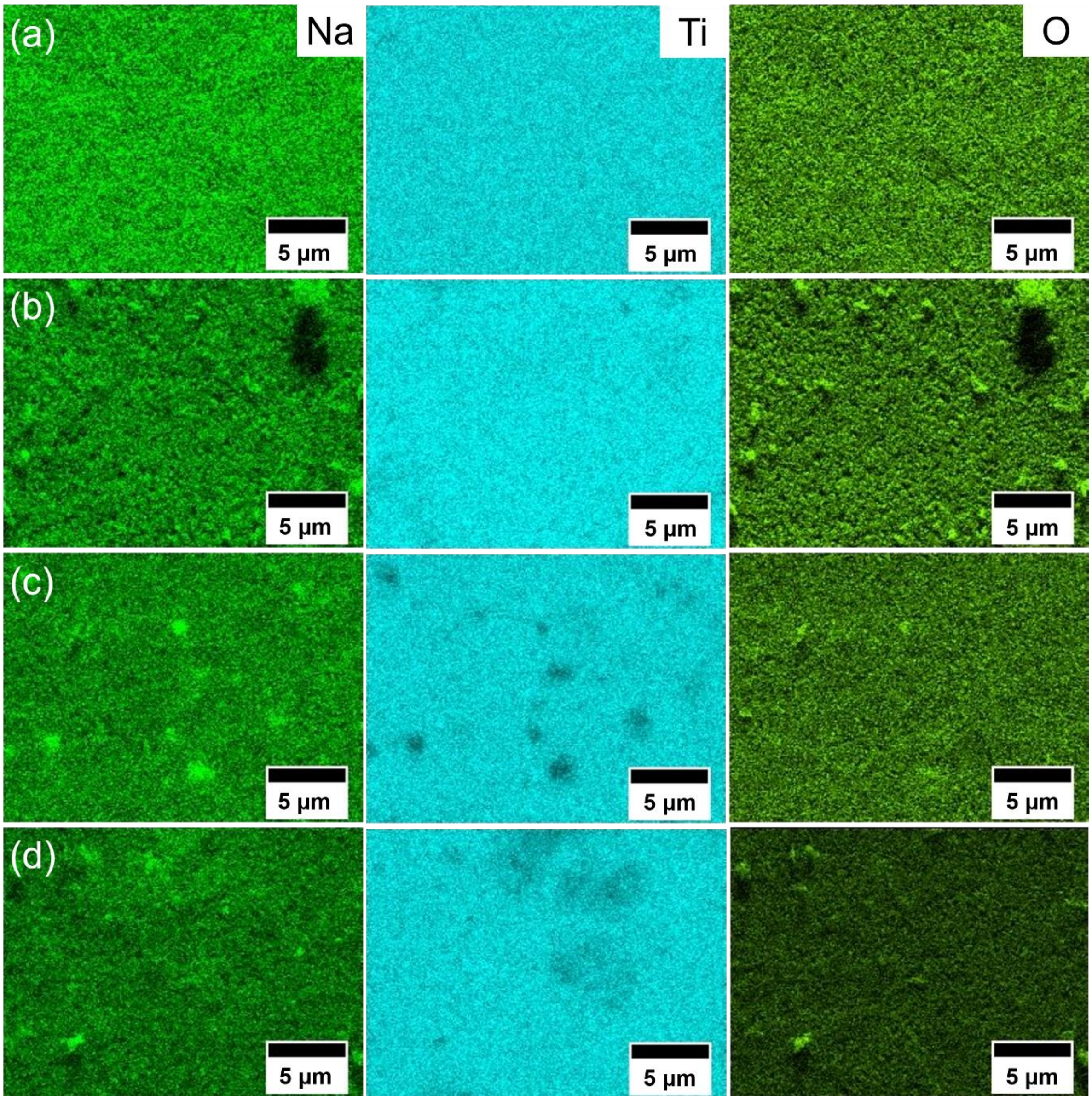

Figure S4. EDX mapping images of elemental Na (left), Ti (middle) and O (right) in samples (a) 0U, (b) 5U, (c) 10U and (d) 20U.

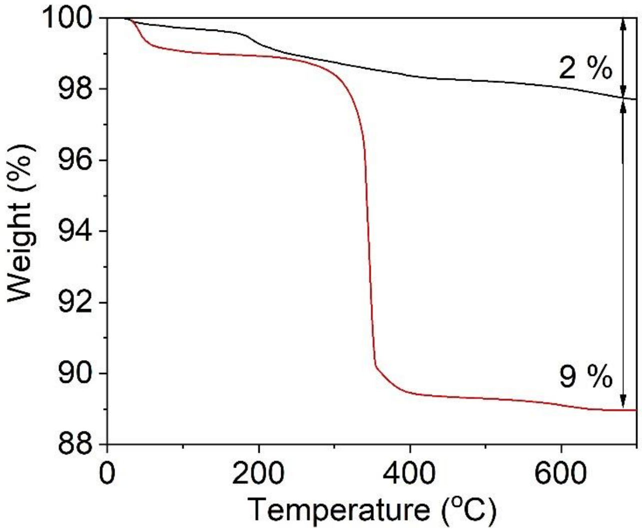

Figure S5. Thermogravimetric analysis data of a typical NTO sample when heated in air, from ambient temperature to 700 °C using a heating ramp of 10 °C min^{-1}. Bare NTO and carbon coated NTO are shown in black and red, respectively.

.

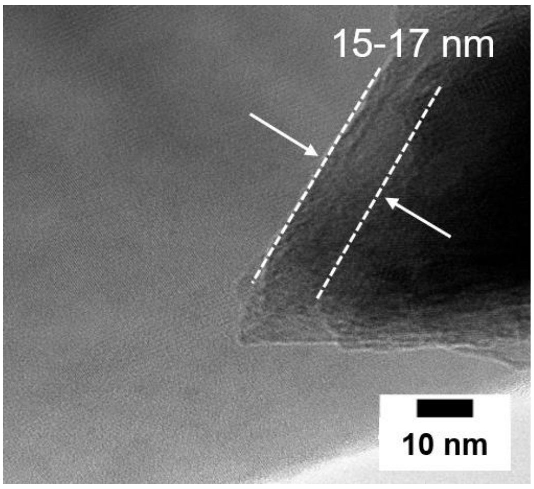

Figure S6. HRTEM image of a typical carbon coated NTO sample at the pristine state. The white arrows indicate the thickness of the carbon layer on the surface of the NTO particle.

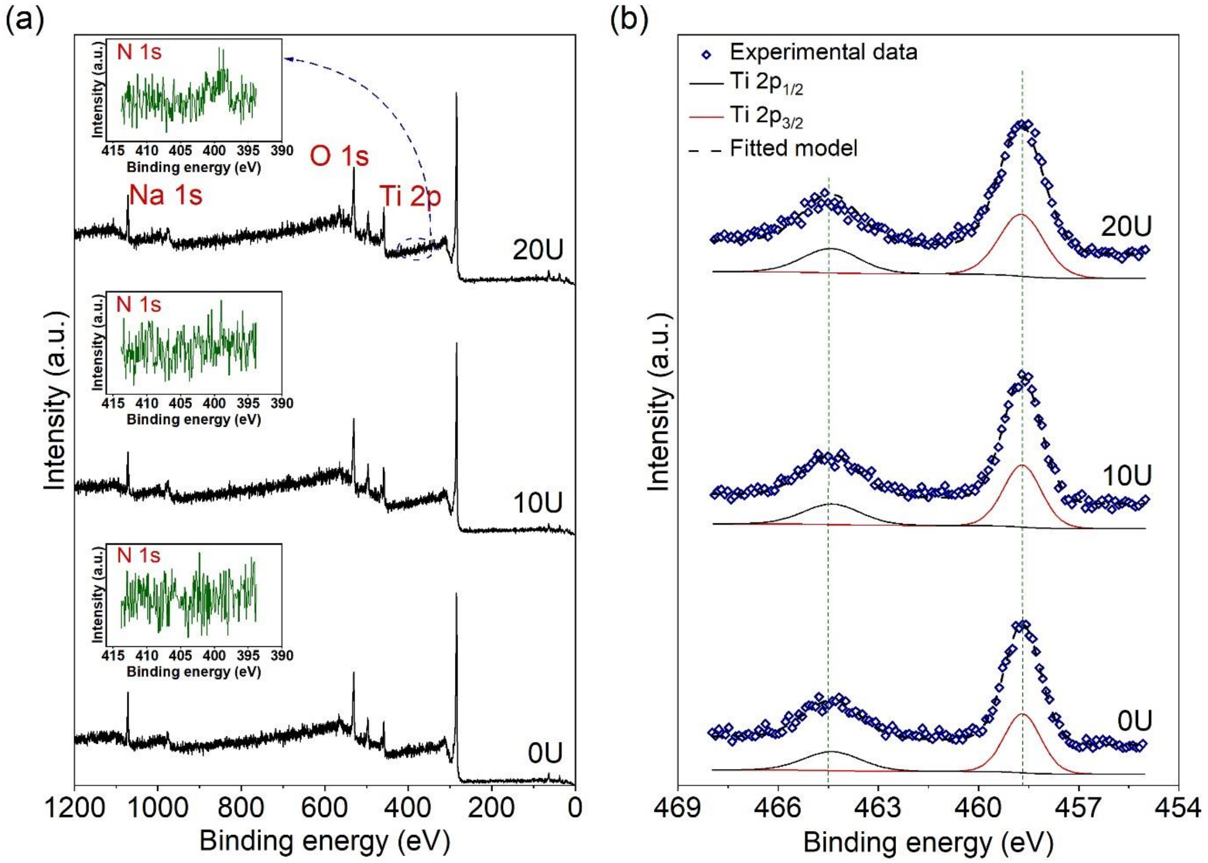

Figure S7. (a) XPS survey of samples 0U, 10U and 20U. Inset: high-resolution N 1s spectra of each sample. (b) High-resolution Ti 2p spectra of samples 0U, 10U and 20U.

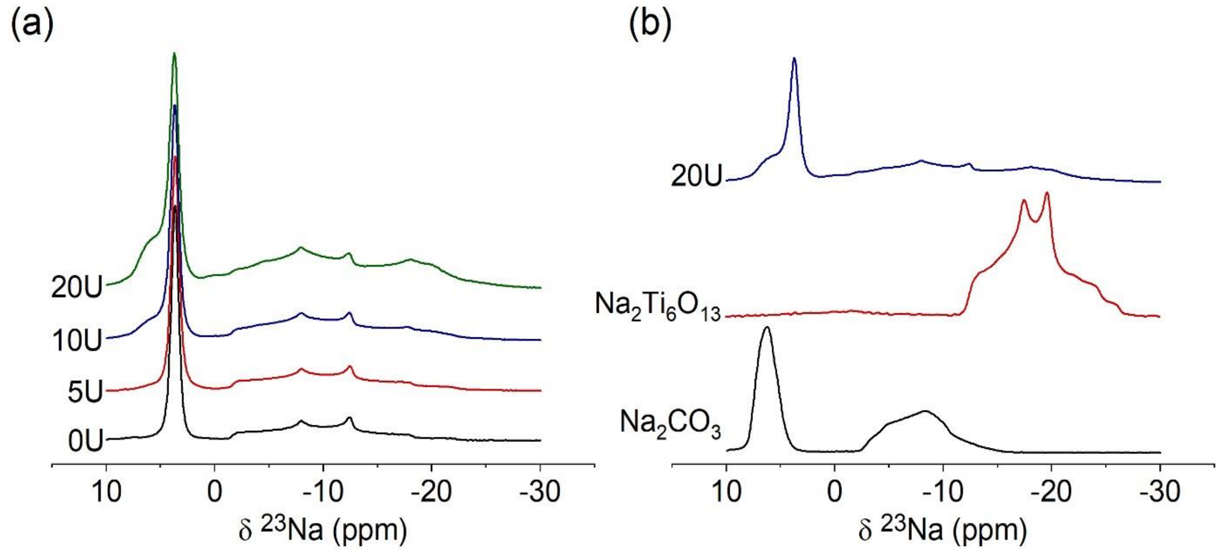

Figure S8. ^{23}Na MAS NMR spectra (ν = 10 kHz) of (a) samples 0-20U and (b) 20U, $Na_2Ti_6O_{13}$ and Na_2CO_3.

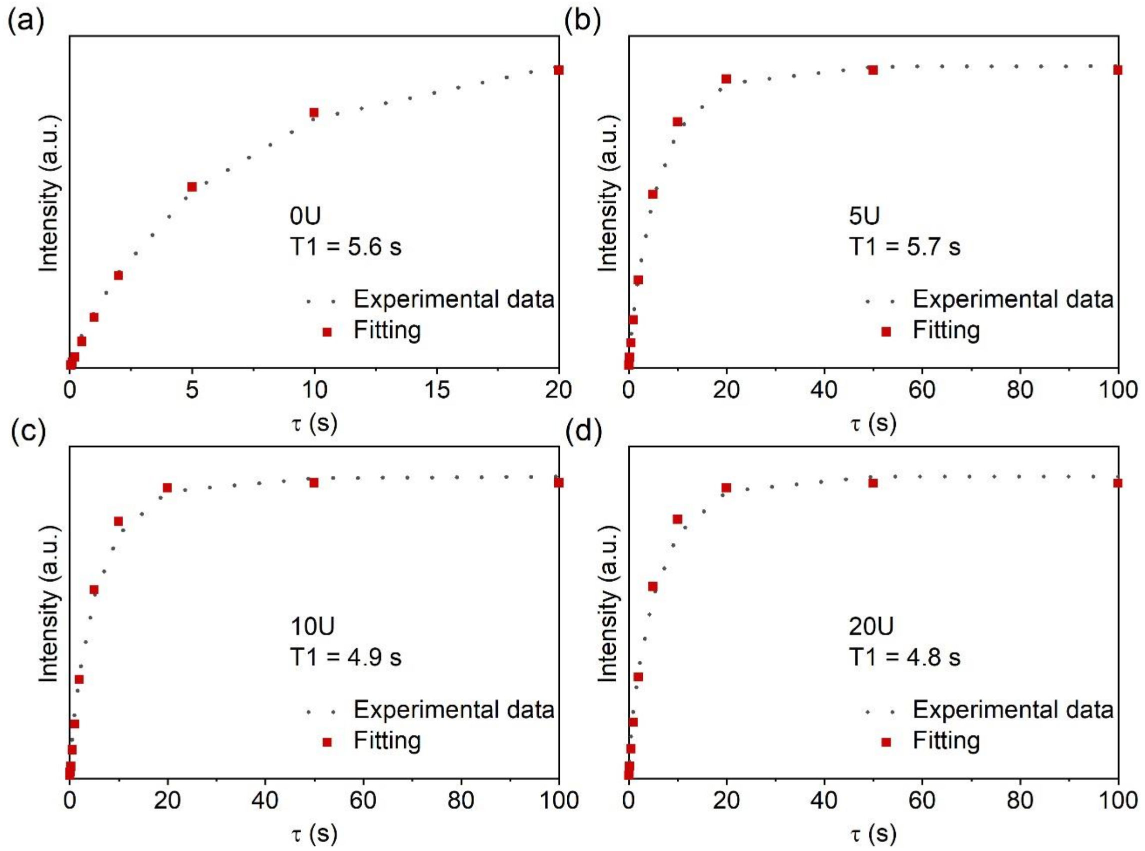

Figure S9. ^{23}Na spin-lattice relaxation time (T_1) of samples: a) 0U, b) 5U, c) 10U and d) 20U, obtained from saturation recovery experiments. Experimental data are fitted to $I(t) = I_0[1 - \exp(-t/T1)]$, where I is the intensity, t is the time (s) and $T1$ is the spin-lattice relaxation time (s).

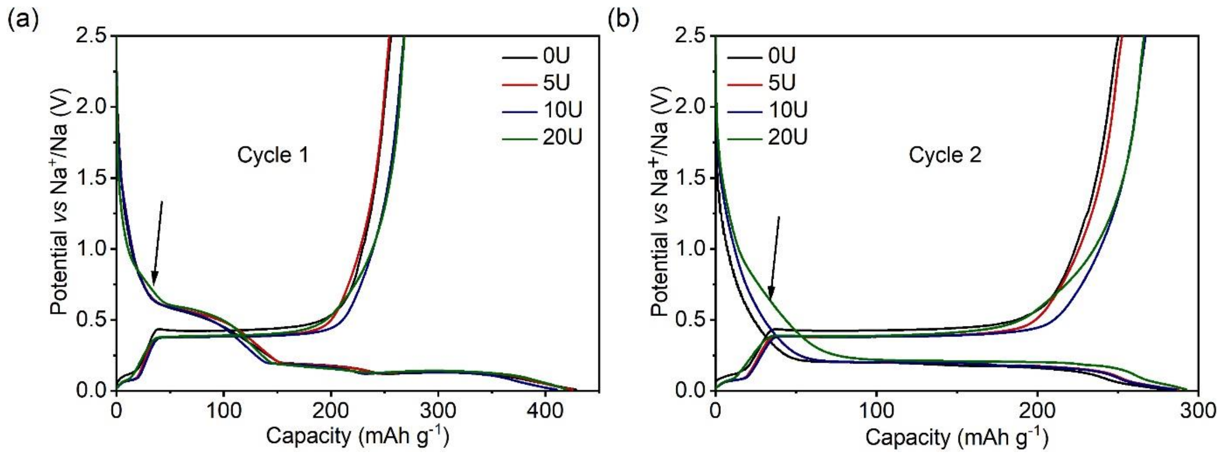

Figure S10. Galvanostatic charge/discharge voltage profiles of 0-20U electrodes in the voltage range 0.01-2.5 V vs. Na^+/Na at 0.1C in (a) cycle 1 and (b) cycle 2. The arrows indicate the plateau corresponding to the insertion of Na^+ into the $Na_2Ti_6O_{13}$ structure.

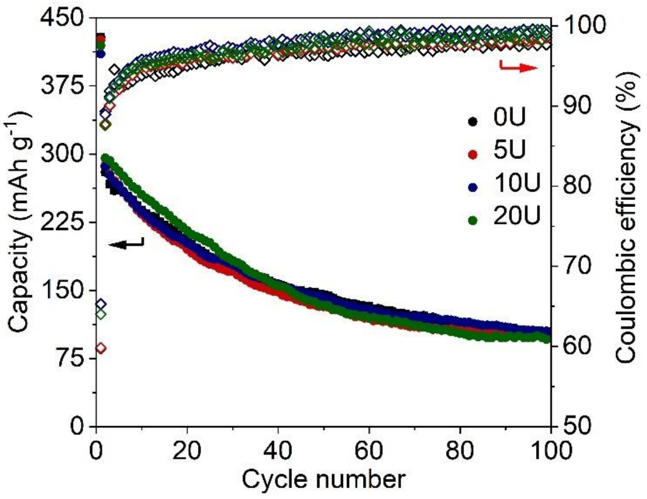

Figure S11. Long-term cycling performance and coulombic efficiencies of samples 0-20U in the voltage range 0.01-2.5 V vs. Na^+/Na at 0.1 C.

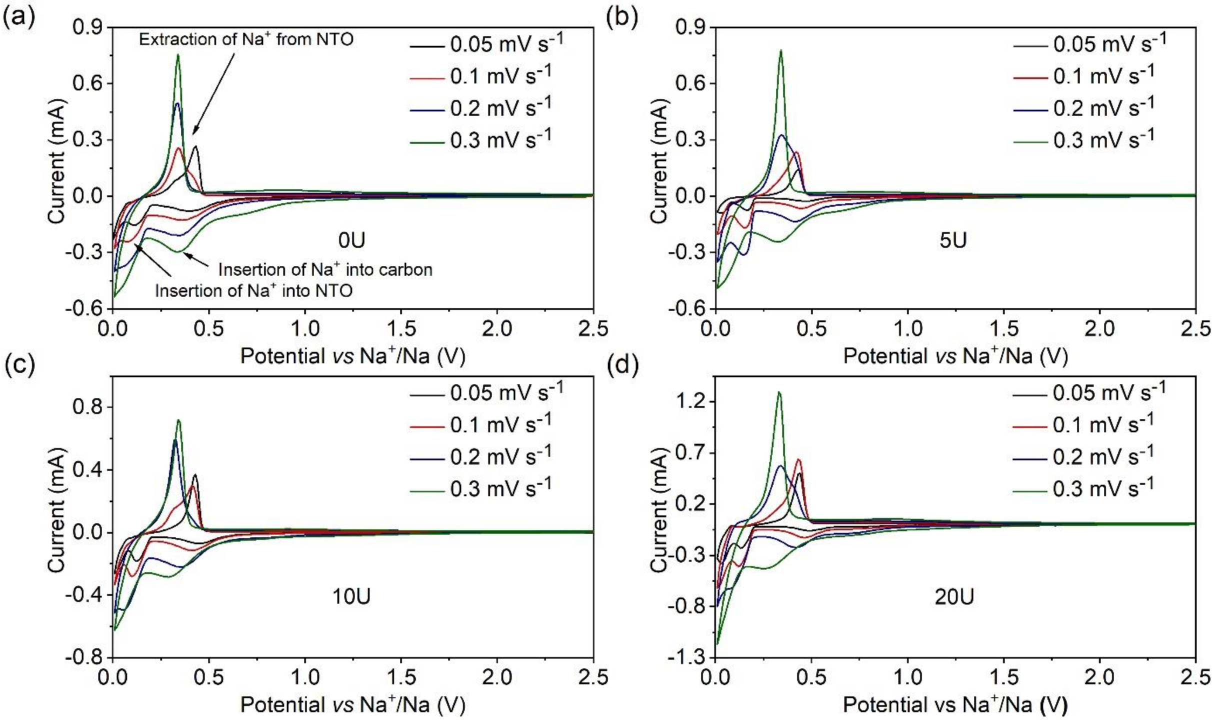

Figure S12. Cyclic voltammograms of (a) 0U, (b) 5U, (c) 10U and (d) 20U electrodes at different scan rates in the voltage range 0.01-2.5 V vs. Na^+/Na.

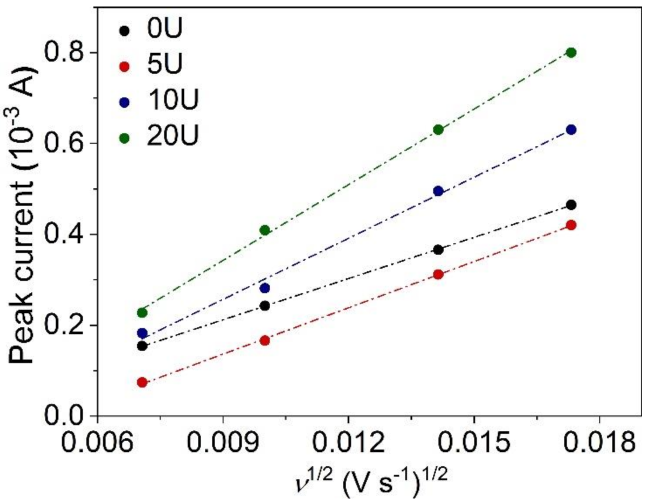

Figure S13. Plot of the cathodic peak current vs. the square root of the scan rate of 0-20U electrodes obtained from the CV measurements ran at different scan rates during the first reduction process (Figure S12).

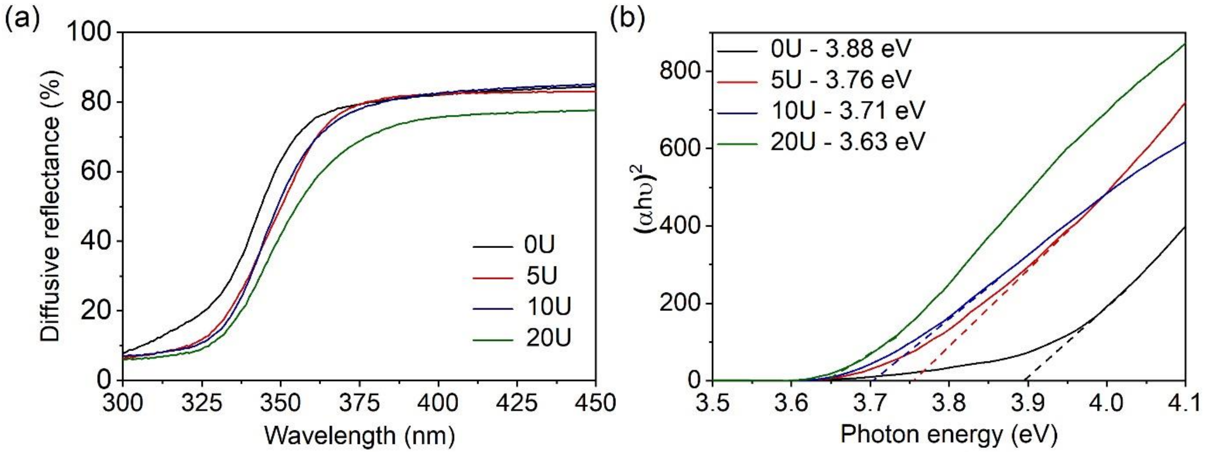

Figure S14. (a) Ultraviolet-visible diffusive reflectance spectra of samples 0-20U in the range 300-450 nm and (b) Tauc plots obtained from the data in (a). Calculated bandgap energies are shown.

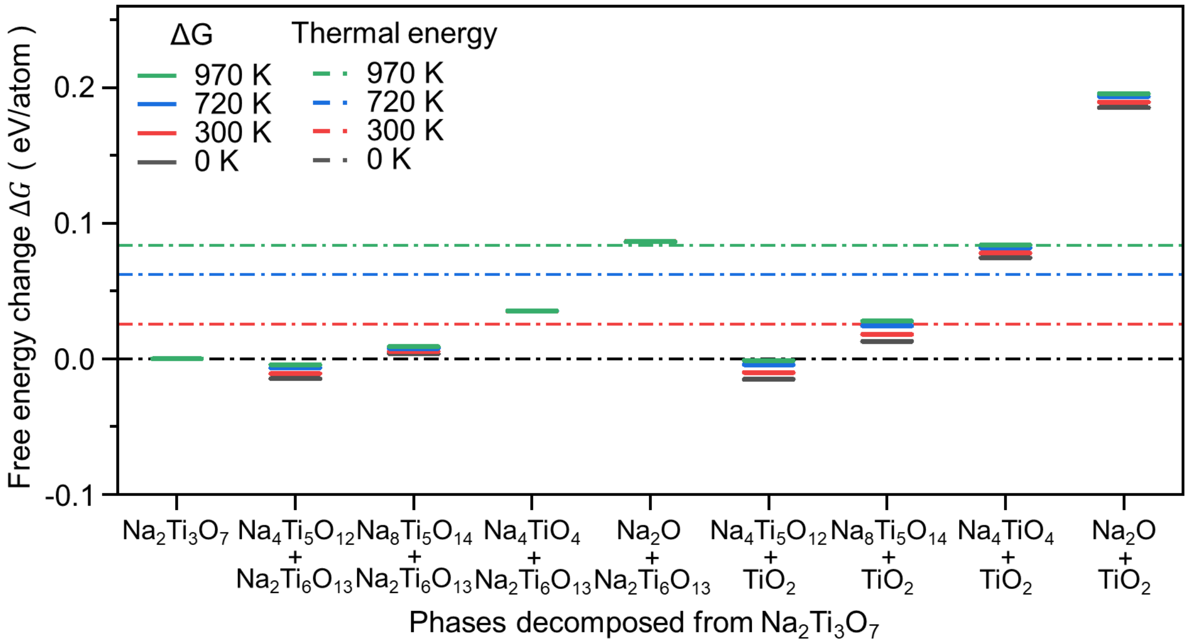

Figure S15. Gibbs free energy changes calculated for various decomposition reactions at 0, 300, 720 and 970 K. Only phases lying on the tie line between Na_2O and TiO_2 are considered. Red, blue and green dashed lines denote the thermal vibration energies imposed at 300, 720 and 970 K, respectively. Note that the majority of ΔG values are located below the thermal vibration energies, indicating that most decomposition processes are expected to be feasible during heat treatment.

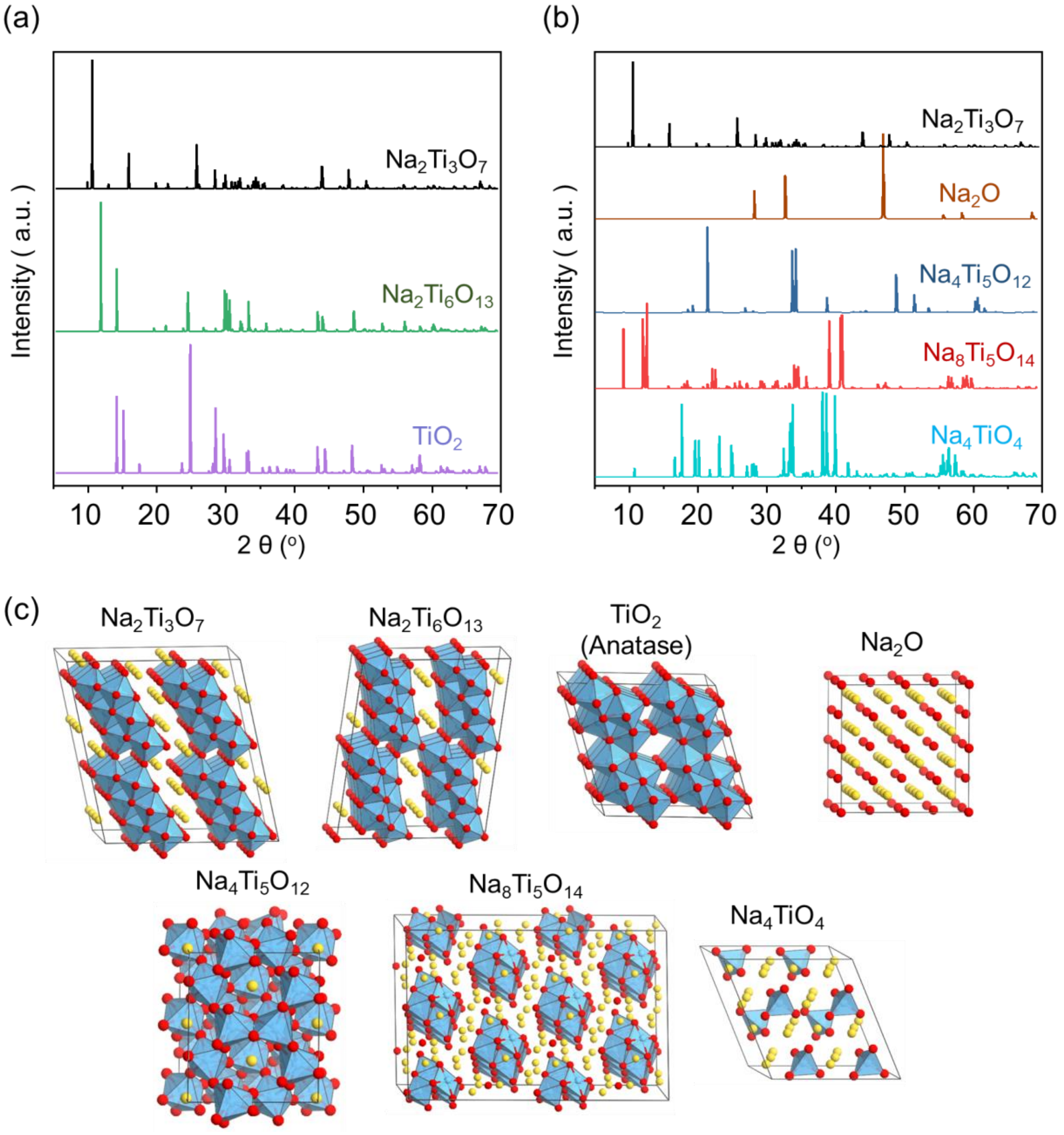

Figure S16. Comparison of the calculated powder X-ray diffraction data of $Na_2Ti_3O_7$ with: (a) $Na_2Ti_6O_{13}$ and TiO_2; and (b) Na_2O, $Na_4Ti_5O_{12}$, $Na_8Ti_5O_{14}$, and Na_4TiO_4. (c) Relaxed atomic configurations of $Na_2Ti_3O_7$, $Na_2Ti_6O_{13}$, TiO_2, Na_2O, $Na_4Ti_5O_{12}$, $Na_8Ti_5O_{14}$ and Na_4TiO_4. All the atomic structures are presented as supercell structures to display the structural similarity between $Na_2Ti_3O_7$ and $Na_2Ti_6O_{13}$.

List of Tables

Table S1. List of unit cell parameters of primitive $Na_xTi_yO_z$ structures relaxed using PBEsol and HSE06 functionals. *k*-point grids used in relaxations are also given for reference. The lattice parameters *a*, *b* and *c* are given in Å, whereas angles *α*, *β* and *γ* are given in °. Conventional structures and experimentally measured values are also shown for comparison.

Phase		*a*	*b*	*c*	*α*	*β*	*γ*	*k*-grids
$Na_2Ti_3O_7$	HSE06	9.12936	3.79883	8.52397	90	101.559	90	2×5×2
	PBEsol	9.10759	3.81039	9.10759	90	101.681	90	
	Exp. *	9.1245(1)	3.79999(5)	8.5624(1)	90	101.592(1)	90	-
$Na_2Ti_6O_{13}$	HSE06	7.795739	7.795739	9.191118	81.190	98.810	152.225	5×5×2
	HSE06 **	15.12283	3.74371	9.17649	90	98.849	90	
	PBEsol	7.803920	7.803920	9.20601	81.220	98.780	152.173	
	PBEsol **	15.06756	3.75476	9.18808	90	98.677	90	
	Exp. [#1]	15.11	3.7467	9.1674	90	99.056	90	-
Na_2O	HSE06	3.863131	3.863131	3.863131	60	60	60	10×10×10
	PBEsol	3.868412	3.868412	3.868412	60	60	60	
Na_2O_2	HSE06	6.123928	6.123928	4.421959	90	90	120	4×4×4
	PBEsol	6.109550	6.109550	4.397835	90	90	120	
NaO_2	HSE06	2.810633	4.573949	5.512361	90	90	90	6×5×4
	PBEsol	3.015530	4.387080	5.511727	90	90	90	
Na_4TiO_4	HSE06	5.721647	5.735648	8.649622	81.866	71.408	68.396	4×3×2
	PBEsol	5.701175	5.712476	8.616202	81.828	71.410	68.463	
$Na_8Ti_5O_{14}$	HSE06	8.332121	8.520873	10.50600	104.17	102.174	112.532	3×3×2
	PBEsol	8.346300	8.534702	10.51418	104.18	102.231	112.530	
$Na_4Ti_5O_{12}$	HSE06	5.312930	5.312931	9.549865	90	90	120	4×4×2
	PBEsol	5.309614	5.309614	9.540872	90	90	120	
$NaTi_2O_4$	HSE06	2.926625	9.265712	10.76361	90	90	90	6×2×2
	PBEsol	2.914181	9.230307	10.72767	90	90	90	
$NaTi_5O_{10}$	HSE06	8.385106	9.428581	10.04654	81.619	67.362	84.434	3×2×2
	PBEsol	8.388806	9.449646	10.04676	81.972	67.536	84.738	
TiO_2	HSE06	6.377643	6.377643	6.543056	73.903	106.097	145.850	7×7×4
	PBEsol	6.398637	6.398637	6.545064	73.859	106.141	145.806	
Ti_3O_5	HSE06	5.192947	5.192947	9.469284	88.326	91.674	137.180	6×6×3
	PBEsol	5.198695	5.198695	9.210697	89.009	90.991	136.209	
Ti_2O_3	HSE06	5.382135	5.382135	5.382134	57.497	57.497	57.497	7×7×7
	PBEsol	5.486210	5.486210	5.486210	54.895	54.895	54.895	
TiO	HSE06	4.962917	4.962917	2.857698	90	90	120	6×6×8
	PBEsol	4.981719	4.981719	2.838221	90	90	120	
Ti_2O	HSE06	2.921846	2.921846	4.771734	90	90	120	9×9×5
	PBEsol	2.946182	2.946182	4.734903	90	90	120	
Ti_3O	HSE06	5.073943	5.073943	9.408729	90	90	120	7×7×4
	PBEsol	5.090587	5.090587	9.374996	90	90	120	
Ti_6O	HSE06	5.045268	5.045268	9.373804	90	90	120	5×5×3
	PBEsol	5.040463	5.040463	9.360071	90	90	120	

* Present study; ** Conventional structure[2]

Table S2. Selected crystallographic data of pristine NTO (0U) obtained from the Rietveld fit of the NTO $P12_1/m1$ structure model (ICSD 250000) against PXRD data at room temperature (Fig. S2).

**Atom**	**Wyckoff position**	***x***	***y***	***z***	**Occupancy**	**100 x U_{iso} (Å^2)**
**Na1**	2*e*	0.5943(7)	0.25	0.6758(7)	1	1.32(5)
**Na2**	2*e*	0.1576(7)	0.25	0.4973(7)	1	1.32(5)
**Ti1**	2*e*	0.9859(4)	0.25	0.1476(4)	1	0.09(5)
**Ti2**	2*e*	0.6774(3)	0.25	0.2488(3)	1	0.09(5)
**Ti3**	2*e*	0.2793(3)	0.25	0.0285(3)	1	0.09(5)
**O1**	2*e*	0.185(1)	0.25	0.222(1)	1	1.39(9)
**O2**	2*e*	0.461(1)	0.25	0.148(1)	1	1.39(9)
**O3**	2*e*	0.649(1)	0.25	0.437(1)	1	1.39(9)
**O4**	2*e*	0.907(1)	0.25	0.334(1)	1	1.39(9)
**O5**	2*e*	0.752(1)	0.25	0.019(1)	1	1.39(9)
**O6**	2*e*	0.3233(9)	0.25	0.794(1)	1	1.39(9)
**O7**	2*e*	0.037(1)	0.25	0.912(1)	1	1.39(9)

$Na_2Ti_3O_7$ – space group $P12_1/m1$

a = 9.1244(1) Å, b = 3.79996(5) Å, c = 8.5624(1) Å, β = 101.592(1) °

χ^2 = 4.046, R_{wp} = 7.85 %, R_p = 5.32 %

Table S3. Electrochemical performance comparison between 20U and other NTO samples reported in the literature.

Material	Synthesis method	Current density (mA g^{-1})	Discharge capacity (mAh g^{-1}) /cycle number	Coulombic efficiency (%)	Electrolyte composition	Ref.
**NTO microflowers**	Solvothermal	200	100/200	----	1 M $NaClO_4$ in EC:DEC (1:1 v/v) with 5 vol.% FEC	3
**NTO nanotubes@C***	Solvothermal	177	142.2/100	> 99	1 M $NaClO_4$ in EC:DMC (1:1 v/v) with 10 wt.% FEC	4
**F-doped NTO**	Solid-state	20	246.3 /1	≈ 49	1 M $NaClO_4$ in DEC:DMC(1:1 v/v)	5
			125/10	> 99		
**NTO@RHCS nanosheets****	Solvothermal	177	180/10	----	1 M $NaClO_4$ in EC:PC with 5 wt.% FEC	6
**Sheet-like NTO**	Solid-state	17.7	290/1	≈ 56	1 M $NaPF_6$ in EC:DMC (1:1 v/v)	7
**Hydrogenated NTO nanoarrays**	Hydrothermal followed by hydrogenation	177	195/10	> 95	1 M $NaClO_4$ in EC:DMC (1:1 v/v) with 2 wt.% FEC	8
**NTO/$Na_2Ti_6O_{13}$**	Hydrothermal	20	80/100	> 99	1 M $NaClO_4$ in EC:PC (1:1 v/v) with 2 wt.% FEC	9
**NTO NW@CNT@rGO*****	Hydrothermal	177	101/100	≈ 90	1 M $NaClO_4$ in EC:PC (1:1 v/v)	10
		354	97.9/100	≈ 89		
**Microsized NTO**	Solid-state	17.7	205/2	≈ 87	1 M $NaClO_4$ in EC:PC (1:1 v/v)	11
		354	130/2	≈ 70		
**NTO nanoribbon array/graphene foam**	Hydrothermal	100	345.2/1	≈ 59	1 M $NaClO_4$ in EC:DEC (1:1 v/v)	12
**NTO nanowires@CC******	Hydrothermal	177	150/100	≈ 87	1 M $NaClO_4$ in PC with 5 wt.% FEC	13
		354	150/50	≈ 98		
**NTO nanofibers**	Electrospinning and hydrothermal	177	125/100	> 99	1 M $NaClO_4$ in EC:PC (1:1 v/v)	14
**Yb-doped NTO**	Solid-state	177	200/10	----	1 M $NaClO_4$ in EC:DMC (1:1 v/v) with 5 % FEC	15
**NTO nanofibers**	Electrospinning	30	270/2	> 95	1 M $NaClO_4$ in EC:DMC (1:1 v/v)	16
		50	200/5	> 95		
**P-doped NTO**	Hydrothermal	106	250/2	----	1 M $NaClO_4$ in EC:DEC (1:1 v/v) with 5 wt.% FEC	17
**NTO nanotube Arrays**	Hydrothermal followed by heat treatment (NH_3/Ar)	177	245/10	----	1 M $NaClO_4$ in EC:DEC (1:1 v/v)	18
**This work (20U)**	Solid-state followed by heat treatment (H_2/N_2)	17.7	419/1	≈ 64	1 M $NaPF_6$ in EC:DEC (1:1 v/v)	-
			96/100	> 99		
		177	316/1	≈ 64		
			160/100	> 99		
		354	272/1	≈ 66		
			150/100	> 99		

*C - Carbon coating; **RHCS - Red blood cell-like hollow carbon sphere; ***NW - nanowires; CNT - carbon nanotubes; rGO - reduced graphene oxide; ****CC - Carbon cloth

Table S4. Charge-transfer resistance determined from EIS for samples 0U and 20U at different states of charge between 100 MHz and 50 mHz.

	**0U**		**20U**	
**Voltage vs. Na^+/Na (V)**	$\boldsymbol{R_{SEI}}$ **(Ω)**	$\boldsymbol{R_{CT}}$ **(Ω)**	$\boldsymbol{R_{SEI}}$ **(Ω)**	$\boldsymbol{R_{CT}}$ **(Ω)**
**OCV – 2.5**	--	104.2	--	40.0
**1.0**	92.7		53.0	
**0.4**	44.6	28.4	39.8	19.1
**0.2**	48.0	18.1	50.7	13.0
**0.01**	77.4		64.9	

List of Equations

Equation S1

The *g-values* obtained from the EPR spectroscopy data (Fig. 2a) were calculated using the following equation:[19]

$$g = \frac{h \; \nu_c}{\beta \, B_0} \tag{S1}$$

where h is the Planck constant (6.626×10^{-34} Js), β is the Bohr magnetron (9.274×10^{-28} J G^{-1}), ν_c is the microwave frequency (9.87 GHz) and B_0 is the magnetic field. The SI unit of the magnetic field is Tesla (T), which is equivalent to 10^4 Gauss.

Equation S2

The sodium-ion diffusion coefficient, D_{Na^+}, was determined from the CV measurements (Fig. S12) using the Randles-Sevcik equation:[20]

$$i_p = 0.4463\, z\, F\, A\, C \sqrt{\frac{z\, F\, v\, D_{Na^+}}{RT}} \qquad \text{(S2)}$$

where i_p is the peak current (mA), z is the number of exchanged electrons (z = 1 for NTO), F is the Faraday constant (96485 C mol^{-1}), A is the electrode geometric area (cm^2), C is the Na^+ ion concentration in the sodium titanate lattice (0.0114 mol cm^{-3}), v is the scan rate (mV s^{-1}), R is the ideal gas constant (8.314 J K^{-1} mol^{-1}) and T is the absolute temperature (300 K).

Equation S3

The charge carrier density was determined using the following Mott-Schottky relationship:[21]

$$\frac{1}{C_{sc}^2} = \frac{2\left(|V_A - V_{fb}| - \frac{kT}{e}\right)}{\varepsilon\, \varepsilon_0\, e\, N A^2} \qquad \text{(S3)}$$

where C_{sc} is the space charge capacitance; V_A is the applied potential vs. Na^+/Na; V_{fb} is the flat band potential, which is the potential needed to flatten the band bending at the counter electrode/electrolyte interface; k is the Boltzmann constant (1.38 x 10^{-23} J K^{-1}); T is the absolute temperature (300 K); e is the electron charge (1.602 × 10^{-19} C); ε is the dielectric constant of the metal oxide; ε_0 is the permittivity of free space (8.854 × 10^{-12} F m^{-1}); N is the density of acceptors within the space charge region; and A is the electrode area (2.83 cm^2). This relationship is only applicable in the voltage window where the capacity varies linearly with voltage.

References

1. Momma K, Izumi F. VESTA 3 for three-dimensional visualization of crystal, volumetric and morphology data. *J Appl Crystallogr*. 2011;44(6):1272-1276.

2. Wu C, Wu ZG, et al. Insight into the Origin of Capacity Fluctuation of $Na_2Ti_6O_{13}$ Anode in Sodium Ion Batteries. *ACS Appl Mater Interfaces*. 2017;9(50):43596-43602.

3. Anwer S, Huang Y, et al. Nature-Inspired $Na_2Ti_3O_7$ Nanosheets-Formed Three-Dimensional Microflowers Architecture as a High-Performance Anode Material for Rechargeable Sodium-Ion Batteries. *ACS Appl Mater Interfaces*. 2017;9(13):11669−11677.

4. Li M, Xiao X, et al. Carbon coated sodium-titanate nanotube as an advanced intercalation anode material for sodium-ion batteries. *J Alloys Compd*. 2017;712:365-372.

5. Chen Z, Lu L, et al. Effects of F-doping on the electrochemical performance of $Na_2Ti_3O_7$ as an anode for sodium-ion batteries. *Materials*. 2018;11(11):1-8.

6. Chen S, Pang Y, et al. Red blood cell-like hollow carbon sphere anchored ultrathin $Na_2Ti_3O_7$ nanosheets as long cycling and high rate-performance anodes for sodium-ion batteries. *J Mater Chem A*. 2018;6(27):13164-13170.

7. Xie M, Wang K, et al. A facile route to synthesize sheet-like $Na_2Ti_3O_7$ with improved sodium storage properties. *Chem Res Chinese Univ*. 2015;31(3):443-446.

8. Fu S, Ni J, et al. Hydrogenation Driven Conductive $Na_2Ti_3O_7$ Nanoarrays as Robust Binder-Free Anodes for Sodium-Ion Batteries. *Nano Lett*. 2016;16:4544-4551.

9. Guo X, Wu Z, et al. Design and Synthesis of Layered $Na_2Ti_3O_7$ and Tunnel $Na_2Ti_6O_{13}$ Hybrid Structures with Enhanced Electrochemical Behavior for Sodium-Ion Batteries. *Adv Sci*. 2018;5(9):1800519.

10. Li Z, Ye S, et al. Free-Standing Sandwich-Structured Flexible Film Electrode Composed of $Na_2Ti_3O_7$ Nanowires@CNT and Reduced Graphene Oxide for Advanced Sodium-Ion Batteries. *ACS Omega*. 2017;2(9):5726-5736.

11. Rudola A, Saravanan K, et al. $Na_2Ti_3O_7$: An intercalation based anode for sodium-ion battery applications. *J Mater Chem A*. 2013;1(7):2653-2662.

12. Dong S, Wu L, et al. Self-supported electrodes of $Na_2Ti_3O_7$ nanoribbon array/graphene foam and graphene foam for quasi-solid-state Na-ion capacitors. *J Mater Chem A*. 2017;5(12):5806-5812.

13. Li Z, Shen W, et al. Ultra-long $Na_2Ti_3O_7$ nanowires@carbon cloth as a binder-free flexible electrode with a large capacity and long lifetime for sodium-ion batteries. *J Mater Chem A*. 2016;4(43):17111-17120.

14. Nie S, Liu L, et al. $Na_2Ti_3O_7$/C Nanofibers for High-Rate and Ultralong-Life Anodes in Sodium-Ion Batteries. *ChemElectroChem*. 2018;5(22):3498-3505.

15. Xia J, Zhao H, et al. Lanthanide doping induced electrochemical enhancement of $Na_2Ti_3O_7$ anodes for sodium-ion batteries. *Chem Sci*. 2018;9(14):3421-3425.

16. Ge Y, Zhu J, et al. Fabrication and electrochemical behavior study of nano-fibrous sodium titanate composite. *Mater Lett*. 2017;188:176-179.

17. Liu DS, Jin F, et al. Phosphorus-Doping-Induced Surface Vacancies of 3D $Na_2Ti_3O_7$ Nanowire Arrays Enabling High-Rate and Long-Life Sodium Storage. *Chem - A Eur J*. 2019;25(65):14881-14889.

18. Liu J, Wang Z, et al. Efficient Surface Modulation of Single-Crystalline $Na_2Ti_3O_7$ Nanotube Arrays with Ti^{3+} Self-Doping toward Superior Sodium Storage . *ACS Mater Lett*. 2019;1(4):389-398.

19. Che M, Giamello E. Electron Paramagnetic Resonance. *Stud Surf Sci Catal*. 1990;57(12):B265-B332.

20. Tang K, Yu X, et al. Kinetic analysis on $LiFePO_4$ thin films by CV, GITT, and EIS. *Electrochim Acta*. 2011;56(13):4869-4875.

21. Bondarenko AS, Ragoisha GA. Variable Mott-Schottky plots acquisition by potentiodynamic electrochemical impedance spectroscopy. *J Solid State Electrochem*. 2005;9(12):845-849.
